# Charge delocalization characteristics of regioregular high mobility polymers†
†Electronic supplementary information (ESI) available: All vacuum calculations and coordinate information. See DOI: 10.1039/c6sc01599a



**DOI:** 10.1039/c6sc01599a

**Published:** 2016-09-20

**Authors:** J. E. Coughlin, A. Zhugayevych, M. Wang, G. C. Bazan, S. Tretiak

**Affiliations:** a Center for Polymers and Organic Solids , Department of Chemistry and Biochemistry , University of California Santa Barbara , Santa Barbara , California 93106 , USA; b Skolkovo Institute of Science and Technology , Moscow , 143025 , Russia; c Theoretical Division , Center for Nonlinear Studies (CNLS) , Center for Integrated Nanotechnologies (CINT) , Los Alamos National Laboratory , Los Alamos , New Mexico 87545 , USA . Email: serg@lanl.gov

## Abstract

Controlling the regioregularity among the structural units of narrow bandgap conjugated polymer backbones has led to improvements in optoelectronic properties, for example in the mobilities observed in field effect transistor devices. To investigate how the regioregularity affects quantities relevant to hole transport, regioregular and regiorandom oligomers representative of polymeric structures were studied using density functional theory. Several structural and electronic characteristics of the oligomers were compared, including chain planarity, cation spin density, excess charges on molecular units and internal reorganizational energy. The main difference between the regioregular and regiorandom oligomers is found to be the conjugated backbone planarity, while the reorganizational energies calculated are quite similar across the molecular family. This work constitutes the first step on understanding the complex interplay of atomistic changes and an oligomer backbone structure toward modeling the charge transport properties.

## Abstract 2

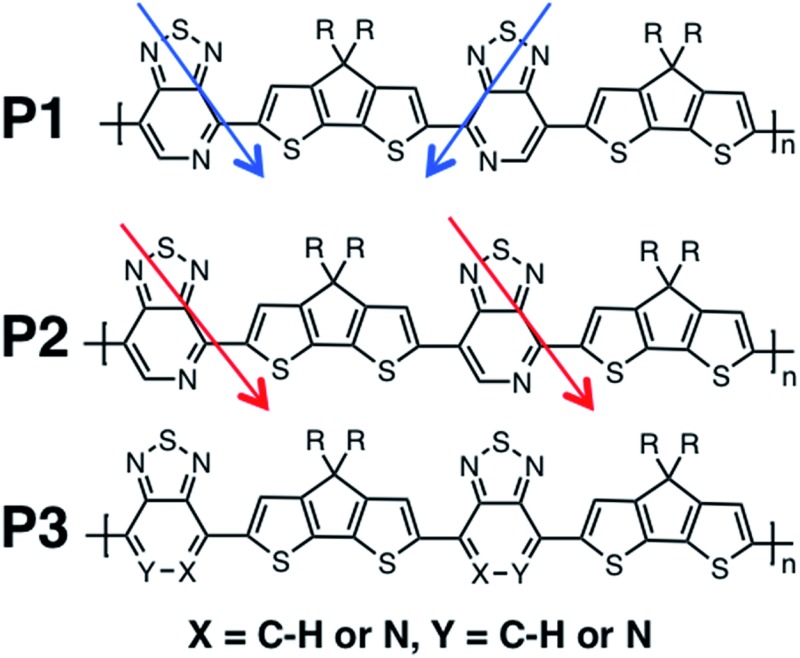
Density Functional Theory modeling examines structural and electronic properties of charge states in the family of narrow bandgap conjugated polymers with controlled regioregularity among the structural units.

Organic semiconductors, such as conjugated polymers and molecules, have been implemented in a wide variety of electronic devices.^1,2^ One particular application involves organic thin film transistors where the conjugated polymer is responsible for charge transport between source and drain electrodes.^3,4^ In order to be commercially viable, the organic layer needs to have a high charge mobility, typically greater than 5 cm^2^ V^–1^ s^–1^.^5^ Several groups have reported high mobilities with oligoacene single crystals and crystalline films.^6–8^ However, because the films have grain boundaries and different grain sizes, the mobility across the film can be inconsistent.^9^ Polymers offer possible advantages because of their ability to be solution processed with large area uniformity and improved mechanical properties.^10^

Recent studies have shown that high hole mobilities (over 20 cm^2^ V^–1^ s^–1^) can be achieved using regioregular conjugated polymers with a backbone comprised of cyclopentadithiophene (CDT) and [1,2,5]thiadiazolo[3,4-*c*]pyridine (also known as pyridyl[2,1,3]thiadiazole, PT) units.^11,12^ These high mobilities are realized when the polymer chains are organized within crystalline fibers and the charge migration predominately occurs along the direction of the fiber. As such, the current understanding is that charge transport occurs predominantly along the polymer chain (either singly or amongst a set of strongly coupled chains). However, it is important to note that the measured transport of charges through the bulk material represents how quickly the holes hop between the chains, as the chains do not span the entirety of the channel of the device. As shown in Fig. 1, the polymers of this study are regioregular and regiorandom, where P1 and P2 have the PT units facing in a regular pattern along the backbone, while P3 has the PT units in random directions, as shown by the arrows in Fig. 1. In previous experimental work, it was found that the hole mobilities between the regioregular arrangement of these CDT and PT units differ by orders of magnitude, with the regioregular polymers having higher hole mobilities.^13^

**Fig. 1:**
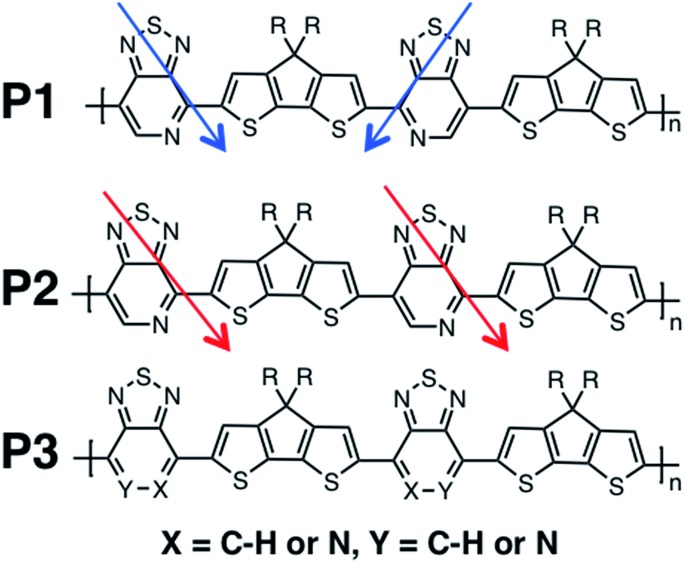
Polymers of interest in this study.

The main mechanism generally accepted for charge transport in organic semiconductors at room temperature is the hopping mechanism of spatially localized charges (polarons).^14,15^ The other mechanism corresponds to band transport, however this applies to a highly ordered system, such as a single crystal of pentacene, at very low temperatures. Thus, both ordering of the material and coupling between electronic and vibrational degrees of freedom ultimately define charge transport in the system.^16^ In the case of hopping transport in disordered systems, the interchain ordering, or morphology, is a deterministic factor that limits transport.^17–19^ Electron–phonon coupling determining the reorganization energies for single electron-transfer events may be a lesser factor, however, its role nevertheless needs to be clarified in relation to the regiochemistry at a single polymer chain level. Moreover, reorganization energies along with electronic couplings provide the necessary microscopic input into theoretical global dispersive transport simulations provided that the morphology of the materials is well understood.

In this contribution, we apply *ab initio* Density Functional Theory (DFT) methods to explore the impact of regiochemistry on the density distribution of the added charge in oligomers that are representative of the polymers of interest. Our emphasis is on cationic species given that the high mobilities in the literature concern hole transport. We demonstrate that by changing the different positions of the units along the backbone, we change the way the oligomer responds to charges. Importantly, the calculations made here are for single chains and are just the start of understanding experimentally-determined hole mobilities.^17^ First the geometry in the ground state between oligomers is evaluated and how this affects charge delocalization along the backbone is discussed. We then examine structural and electronic changes that occur when the oligomer is charged by understanding the similarities and differences of the excess charge locations, bond length alterations, and dihedral differences between the neutral and charged species. Finally, intramolecular reorganization energies and their relationship to the differences in mobility are discussed. What we learned is that the regioregular oligomers tend to adopt more planar structures and have more delocalized spin densities. All three oligomers show a change in the bond dihedrals and bond lengths to accommodate the positive charge, as well as charge accumulation on the CDT unit. The greater regioregularity also leads to a slightly higher intramolecular reorganization energy due to the increased “stiffness” of the polymer backbone.

We calculate the electronic structure of the three oligomers shown in Fig. 2, which correspond to the respective polymers in Fig. 1. Here, groups X and Y in the oligomers are the same as the corresponding polymer (O1 = P1, O2 = P2, O3 = P3). The bulky side-chain R is represented by the truncated group CH_3_. Oligomers O1 and O2, which represent the two regioregular cases P1 and P2, respectively, have 1–5 units in length. Regiorandom O3 has many different PT orientations due to the unknown nature of the variation of the PT unit along the backbone. Therefore, an approximation of the regiorandom 1–5 repeat-unit oligomer was created using a random number generator to arbitrarily determine which way each PT unit was facing. Here we discuss only properties of 5-unit oligomers, whereas additional computational results for smaller systems are summarized in the ESI.† All electronic structure simulations were performed using the Gaussian 09 software package.^20^ The ground state geometries were optimized using the CAM-B3LYP/6-31G** functional and basis sets both in vacuum and solvent. Dielectric medium effects were introduced in the form of the conductor-like polarizable continuum model (CPCM)^20–22^ to model the chlorobenzene solvent, as implemented in the Gaussian 09 software package. This functional/basis set combination has been used in previous studies to describe similar types of donor–acceptor conjugated systems, with fairly good agreement with experimental optical and electronic characterizations.^23^

**Fig. 2:**
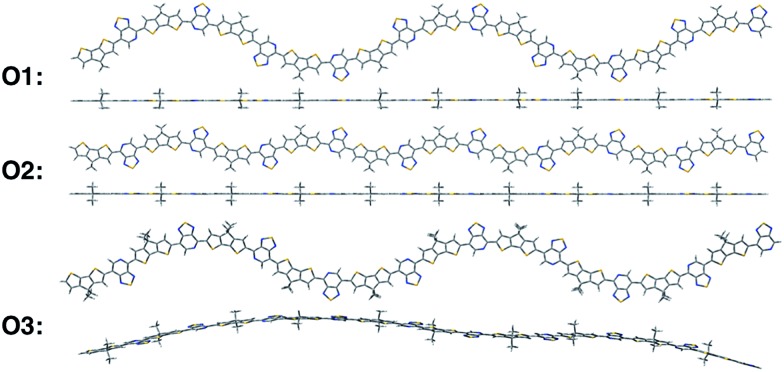
Optimized geometries of O1–O3 in solvent environment.

All oligomers have a lowest energy conformation described by a wave-like, planar shape (Fig. 2). Previous studies of donor–acceptor polymers show the same type of form.^24^ The regioregular PT-containing oligomers O1 and O2 show planar backbones when compared to regiorandom O3. The planarity of the molecules can be influenced by different intramolecular interactions that arise from the heteroatoms in the donor and acceptor fragments. For example, structural units that have multiple heteroatoms, such as PT, favor planarity and stiffness along the backbone of polymers by “locking” adjacent fragments in specific conformations.^25–27^ This feature was illustrated by plotting the difference in energies when the bond between them is rotated, or rotational barrier, between different units.^28,29^ Consequently, the regioregular polymers have their units placed in a configuration that maximizes these intramolecular interactions, while the regiorandom, O3, has a less optimized configuration along the backbone. Overall, the regioregular O1 and O2 are the most planar, while O3 is less planar in both vacuum and solvent conditions.

To gain insights into how the regioregularity of the units along the backbone affect hole delocalization, the spin densities for the cation oligomers were plotted (Fig. 3). In an effort to approximate the properties of a long polymer system, we examined the spin density as a function of repeat units. As shown in the ESI,† the densities became unconstrained by the oligomer ends once a length of five repeat units is reached.

**Fig. 3:**
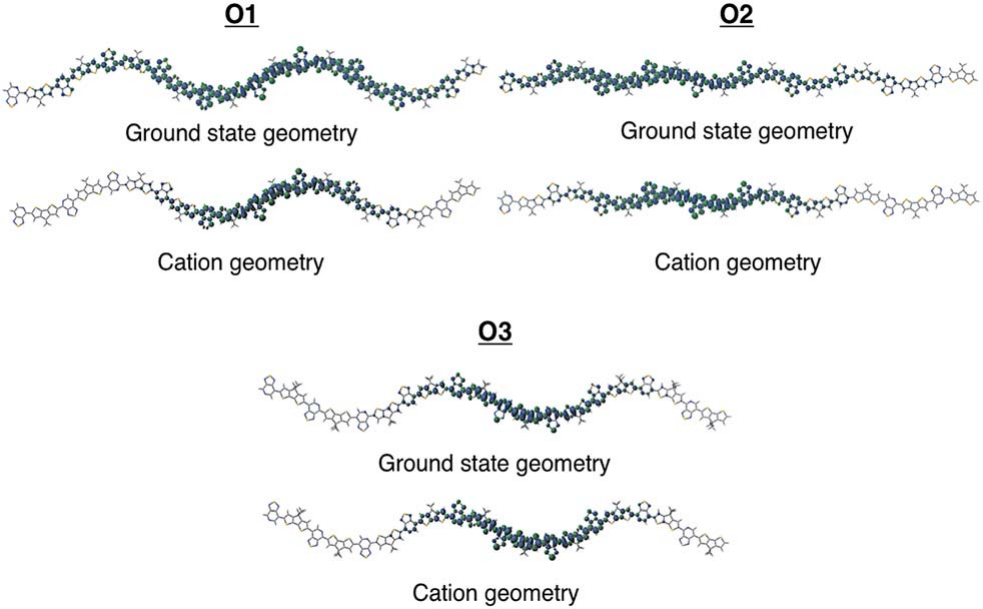
Cation spin density of O1–O3 in the ground state optimized geometry and the cation optimized geometry.

When going from the ground state geometry to the relaxed cation geometry for the cation species, the spin densities for O1 and O2 localize to near the center of the molecule.^30^ This self-trapping develops due to geometric distortions that happen during the relaxation of the cation species. There is also a localization that occurs when comparing the spin density orbitals in vacuum and solvent due to presence of polarization effects introduced by the dielectric medium.^31,32^ The holes are more delocalized for all oligomers in vacuum.^33^ For O3, there is very little change between the different geometries for the cation species, which is most likely due to the distortion of the backbone that is present in both geometries and limits the delocalization length of the positive charge to a small portion of the backbone.

After plotting the spin densities, we investigated in which unit along the backbone the localization of the charges occurs. This can be accomplished by looking at the difference in the natural atomic charge^34^ for each atom between the neutral and charged cationic species, or the “excess charge”, then grouping the atoms together by unit. Natural atomic charges are obtained from natural bond orbital methodology.^34^ Then for each ring along the backbone we are able to plot the “excess charge” and examine how this differs between the different oligomers, as shown in Fig. 4a. For all oligomers in the positively charged state (cation), the CDT structural unit has the highest excess charge. This is not surprising considering the highest occupied molecular orbital (HOMO) for these donor–acceptor type polymers is generally centered on or is the most concentrated around the donor unit, which in our case is the CDT. In contrast, the excess charge for the anion species is on the PT ring, which follows the same trend as the lowest unoccupied molecular orbital (LUMO) for these D–A polymers typically being centered on the electron-accepting unit. These results show that the charge is relatively localized along the backbone in these oligomers, which contrasts other studies for homopolymers, such as polythiophene and polyselenophene in which the charge is delocalized over the whole backbone, even up to long oligomer lengths.^35^ This difference may be traced to the push–pull nature of the electron density, or intramolecular charge transfer, or intramolecular charge transfer, between the donor and acceptor units along the backbone of the oligomers in this study.^36^

**Fig. 4:**
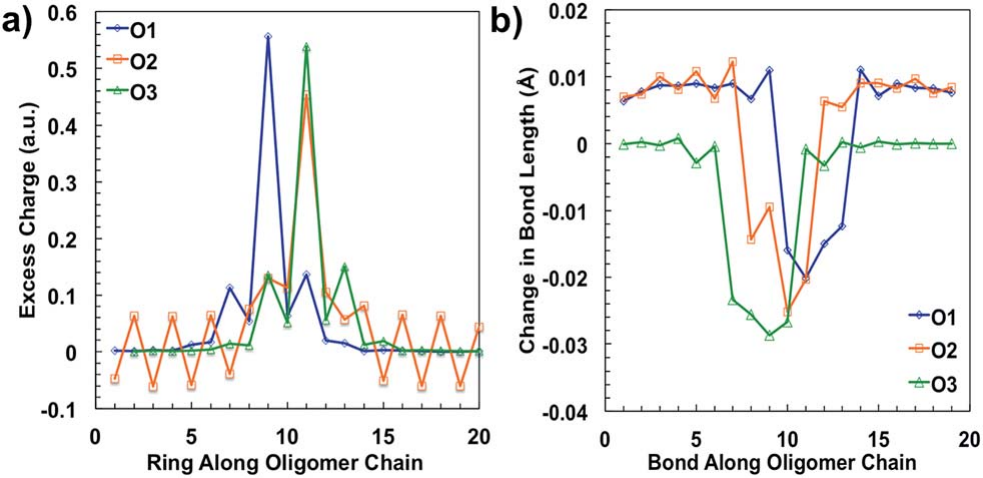
(a) Changes in bond length in O1–O3 from the relaxed ground state geometry to the relaxed cation geometry and (b) excess charge per ring for the cation species of O1–O3 along the backbone, calculated in solvent environment.

When an electron is given to or taken away from the backbone, there are geometric changes that occur to the oligomer.^37^ All three oligomers in the neutral state have an alternating bond length between units along the backbone that is typical for a conjugated system. When an electron is removed from the oligomer, the bond lengths change to accommodate and localize the net positive charge in the areas indicated by the spin densities, similar to what is shown in Fig. 3. As shown in Fig. 4b, the changes in the bond length from the neutral state to the relaxed cation species show this phenomenon. For all oligomers, there is very little change in the bond lengths toward the outer units; however, there is a larger change toward the middle. The bonds for all oligomers shorten towards the center of the oligomer, which is indicative of the spatial changes that occur to accommodate the charge. All three oligomers show that there is a greater change in bond length for the cation species than for the anion (see ESI†), which may indicate that there is less spatial rearrangement needed to accommodate a negative charge. This difference will be discussed further in the intramolecular reorganization section later in this report.

Bond length alteration and changes in bond length are good reflections of the geometric changes that occur in these oligomers, but the dihedral angle changes can also be examined as a good complement to obtain a more complete picture. The dihedral changes are often more subtle than the bond length changes, but are nonetheless indicative of the changes the backbone undergoes to accommodate the charge. For O1 and O2, the dihedrals along the backbone are mostly 0° for the neutral species in solvent, owing to their very planar backbones (Fig. 5). O3 has varied dihedral angles for the neutral species in solvent, with angles up to 20° between some units. When the oligomers become positively charged, there is a large change in the dihedrals for O1 and O2. However, for all oligomers the dihedrals toward the center of the relaxed cation oligomer become more planar to accommodate for the charge.

**Fig. 5:**
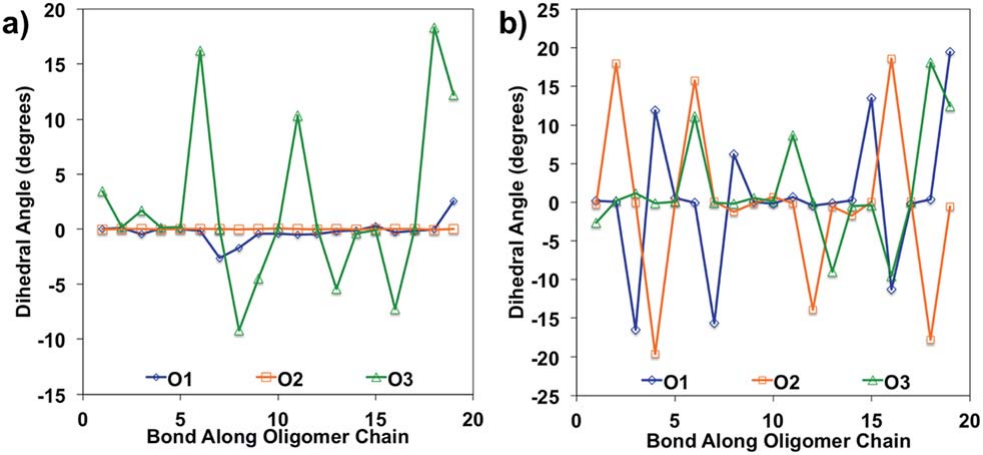
Dihedral angles along the oligomer backbone for (a) neutral oligomers and (b) relaxed cation oligomers, calculated in solvent environment.

After qualitative analysis of the overall geometric and atomistic changes involved in adding and removing electrons, quantification of the changes can give us an overall comparison of the oligomers taking into account all of the evaluations thus far. The quantification of the differences in energies between neutral and charged species (or the intramolecular reorganization energy) is an important parameter in discussing the differences of mobility between polymers. For example, the charge transfer rates in hopping transport^38^ are defined by two parameters according to Marcus theory: the electronic coupling between pairs of electronic sites (such as neighboring polymer chains) and the internal (or intramolecular) reorganization energy.^39^ The electronic coupling parameter is relevant to the orbital overlap and remains outside the scope of this study. The internal reorganization energy quantifies variation of electronic energy due to the geometry changes when an electron is added to or removed from the polymer. There is also the outer reorganization energy that takes into account the changes in polarization of the medium around the polymer. The intramolecular reorganization energy is the sum of two components (*λ*_1_ and *λ*_2_) shown below in eqn (1),1
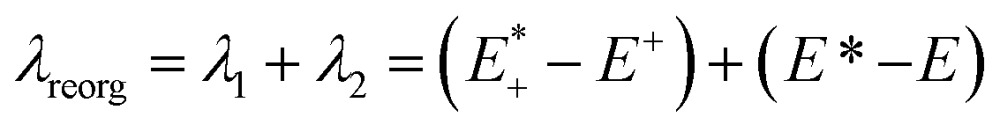
where *λ*_1_ represents an energy decrease between the cation in the neutral species geometry 
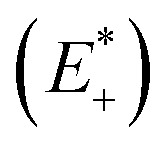
 and the relaxed cation geometry (*E* ^+^). The change in energy between the neutral species in cation geometry (E*) and the relaxed neutral species (*E*) is represented by *λ*_2_.

Reorganization energies calculated using eqn (1) are summarized in Table 1. Overall, one observes for all oligomers a decrease in the reorganization as the number of repeat units increases (see ESI†). Both *λ*_1_ and *λ*_2_ quantities converge rapidly with the number of repeat units. Subsequently, to attain the saturation limit, it is sufficient to consider an oligomer of a size larger than the polaron size. Oligomers shown in Fig. 3 satisfy this condition. Moreover, the reorganization energy decreases when going from vacuum to solvent. This has to do with the stabilization of charges along the backbone due to the polarization effects of the dielectric medium and is generally considered when the outer reorganization energy discussed earlier is evaluated. However, for O1, the reorganization energy slightly increases and this is due to the fact that the *λ*_1_ for this oligomer is increased in the solvent calculations. For O2, there is a small increase in *λ*_1_ as well, however the large decrease in *λ*_2_ leads to a lower overall energy for *λ*_reorg_ when going from vacuum to solvent. Comparing the *λ*_reorg_ across the oligomers, the regioregular oligomers have the highest reorganization energies. O3 has the lowest reorganization energy overall and we attribute this to the increased flexibility of the backbone because it has less intramolecular “locks”, due to the incomplete matching of heteroatoms that leads to these favorable interactions.

**Table 1:** Calculated intramolecular reorganization energies for the cationic species of O1–O3

Oligomer	Medium	*λ*_1_ (meV)	*λ*_2_ (meV)	*λ*_total_ (meV)
O1	Vacuum	248	294	542
	Solvent	330	221	551
O2	Vacuum	243	314	557
	Solvent	244	242	486
O3	Vacuum	260	309	569
	Solvent	218	226	444

Within this study, we were able to isolate and assess the different intramolecular changes that occur upon removal of an electron in the donor–acceptor oligomers of interest. The differences between the regioregular and regiorandom oligomers lie mostly in the overall geometry and stiffness of the backbone. Regioregular O1 and O2 have a more planar backbone than the regiorandom O3. Indeed, O3 exhibits backbone distortion due to the lack of stronger intramolecular “locks”. Delocalized spin densities are linked into the more ordered and more conjugated ground state geometries of O1 and O2, compared to that of O3. The bond length and dihedral angle changes between units from neutral to cation species are consistent across all oligomers, with a bond shortening and dihedral planarization where the localization of the charge occurs. For all oligomers, the excess positive charge is localized around the CDT donor unit and the excess negative charge is localized around the PT acceptor unit.

Overall, the intramolecular reorganization energy computed for the oligomers does not seem to provide the full picture of how the regioregularity impacts hole mobility. Even though the reorganization energy in O3 is slightly smaller compared to that in O1 and O2, the experimental mobilities in the latter are higher. This is relevant to the chain ordering in the regioregular case of O1 and O2, enabling efficient intrachain charge transport. Moreover, very delocalized charge densities in the charged states at the ground state geometry (*i.e.* ‘hot’ charge) observed in O1 and O2, compared to O3, likely facilitate better electronic coupling and faster transport of charges even before nuclear relaxation occurs toward band-transport model.

The fact that the calculated reorganization energies are formally uncorrelated with macroscopic charge mobilities, may also be explained with conjecture that chain conformations and disorder may significantly affect the internal reorganization energies. To address this issue, we use a tight-binding model to rigorously prove that the electronic structure of isolated polymers at optimized planar geometry is insensitive to regiochemistry. The summary of tight-binding calculations are included the ESI.† Thus, internal reorganization energy is a robust descriptor of a given molecular material, which can be obtained from consideration of equilibrium structures of the single chains as demonstrated in our simulations.

This result outlines the complexity of the issue of being able to *a priori* predict how changes in chemical structure that differ only with respect to orientation of structural units affect the mobility of the polymers. Consistent with previous studies,^17–19^ the differences in mobility likely lie in the way the polymers pack together and the electronic coupling between the chains in the bulk. This coupling can only be assessed with precise geometrical information on the relative distances, orientation and translations between chains; information that is not available at this point in time.
