# Supplementary material for: Charge delocalization characteristics of regioregular high mobility polymers
Source: Chem Sci. 2016 Sep 20;8(2):1146–51. doi: 10.1039/c6sc01599a (PMC5369528; doi:10.1039/c6sc01599a)
Supplement: Supplementary file 1 [file SC-008-c6sc01599a-s001.pdf]

Supporting Information

Charge Delocalization Characteristics of High Mobility Regioregular Polymers

Jessica E. Coughlin[a], Andriy Zhugayevych[b], Ming Wang[a], Guillermo C. Bazan[a], Sergei Tretiak[c,b,a]*
[a] Center for Polymers and Organic Solids, Department of Chemistry and Biochemisry, University of California Santa Barbara, Santa Barbara, California, 93106, USA
[b] Skolkovo Institute of Science and Technology, Moscow, Russia, 143025
[c] Theoretical Division, Center for Nonlinear Studies (CNLS) and Center for Integrated Nanotechnologies (CINT), Los Alamos National Laboratory, Los Alamos, New Mexico 87545, USA

Contents

Full Calculation Details	1
Optimized Geometries	2
Spin Density Plots in Vacuum	2
Bond Lengths in Vacuum	3
Bond Length Changes	3
Bond Dihedral Changes	4
Excess Charges Plot in Vacuum	4
Absolute Energies of Oligomers	5
Cation Oligomer (n=1,2,4,5) Reorganization Energies	7
Cartesian Coordinates for n=5 Oligomers	7
First principle tight binding modeling of planar polymers	9

Full Calculation Details

All the work was done using density functional theory with the Gaussian09 software suite. The neutral and charged geometry optimizations were completed using the CAM-B3LYP functional with the 6-31g** basis set. The conductor-like polarizable continuum model and chlorobenzene solvent as implemented in the Gaussian 09 software package were used for solvent-based calculations. Excess charges were calculated using the natural population analysis in the NBO suite of calculations included in Gaussian09. The atom charges in the neutral species were subtracted from the charged and the differences was added up according to what atoms along the backbone were included in each unit, either CDT or PT/BT. The internal reorganization energies were completed using the following equation:

$$\lambda = \lambda_1 + \lambda_2 = (E_+^* - E_+) + (E^* - E)$$

where the lambda 1 is the difference in the charged species and lambda 2 is the difference in the neutral species.

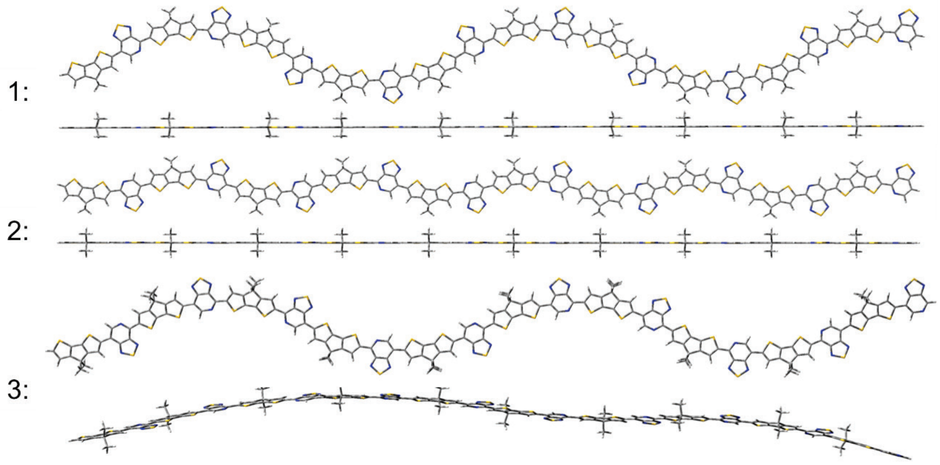

Figure S1. Geometry optimization in vacuum.

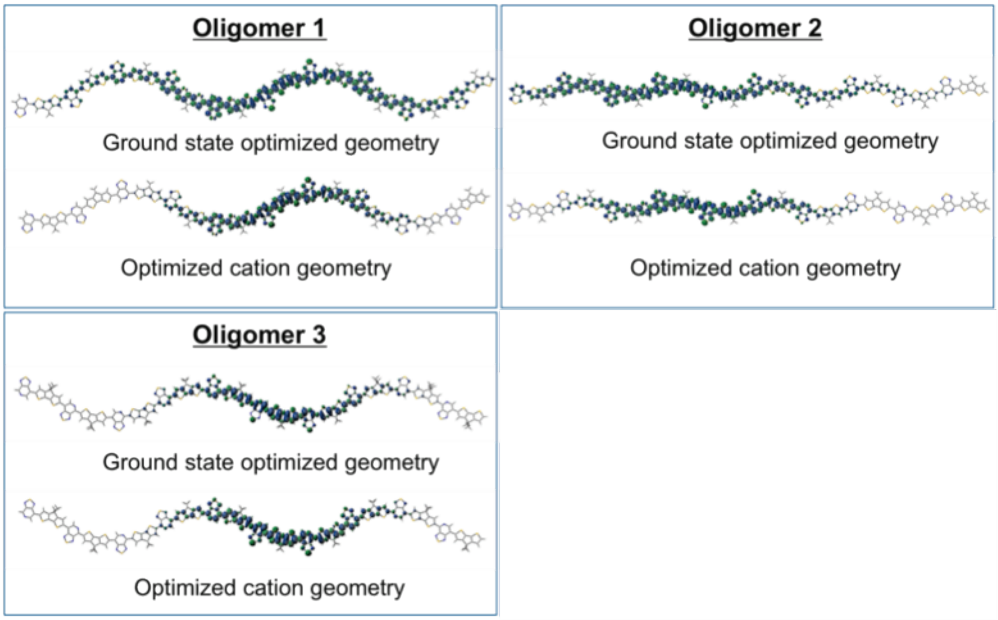

Figure S3. Cation spin density plots in vacuum.

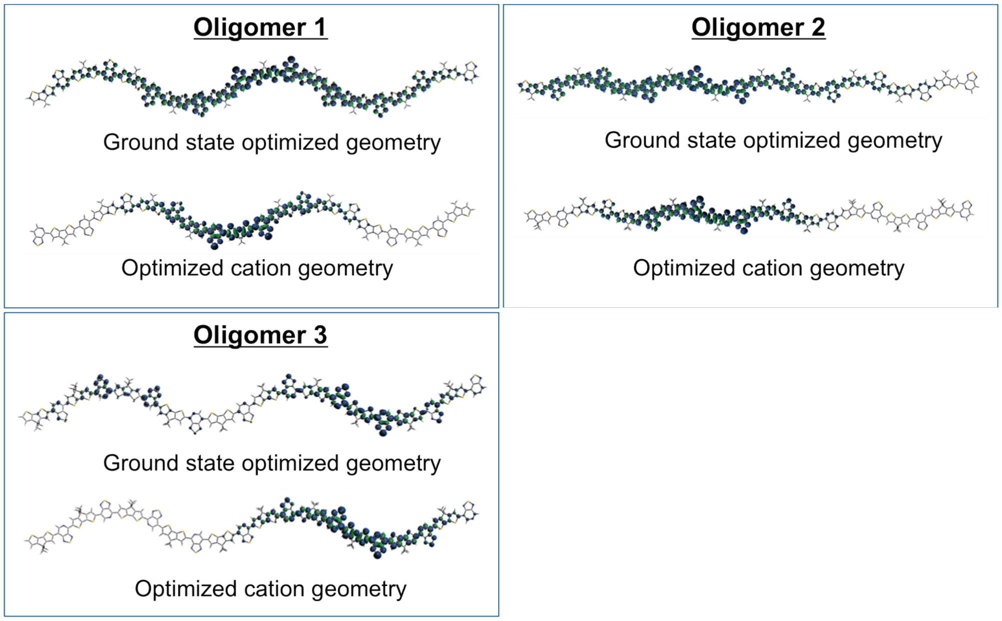

Figure S4. Anion spin density plots in vacuum.

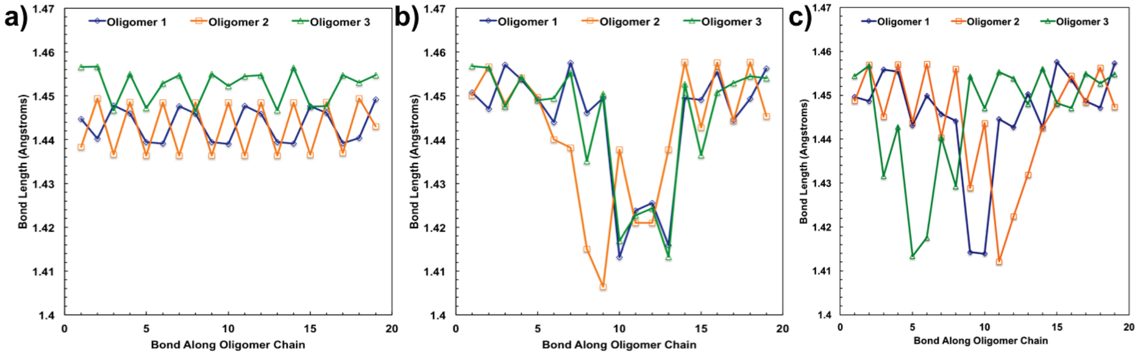

Figure S4. Bond lengths along the backbone for oligomers in vacuum for (a) the neutral species, (b) the cation species, and (c) the anion species.

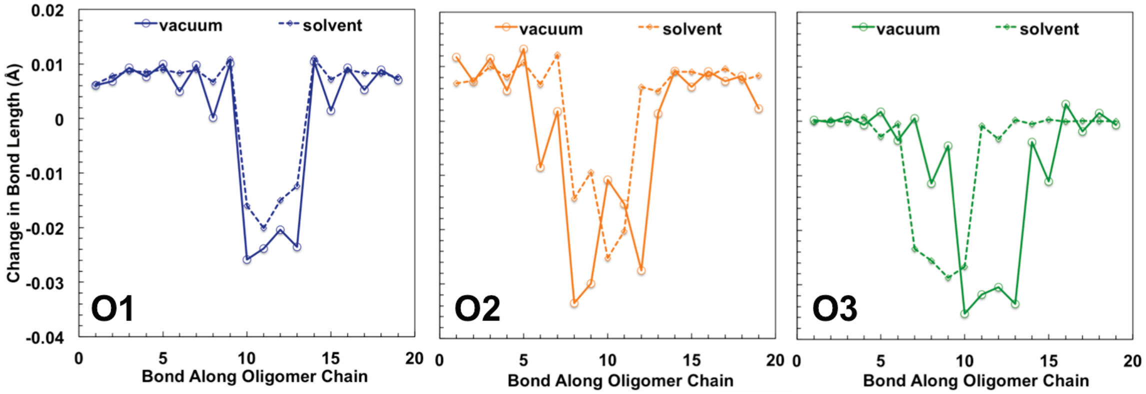

Figure S5. Bond lengths changes for oligomers in vacuum (cation species).

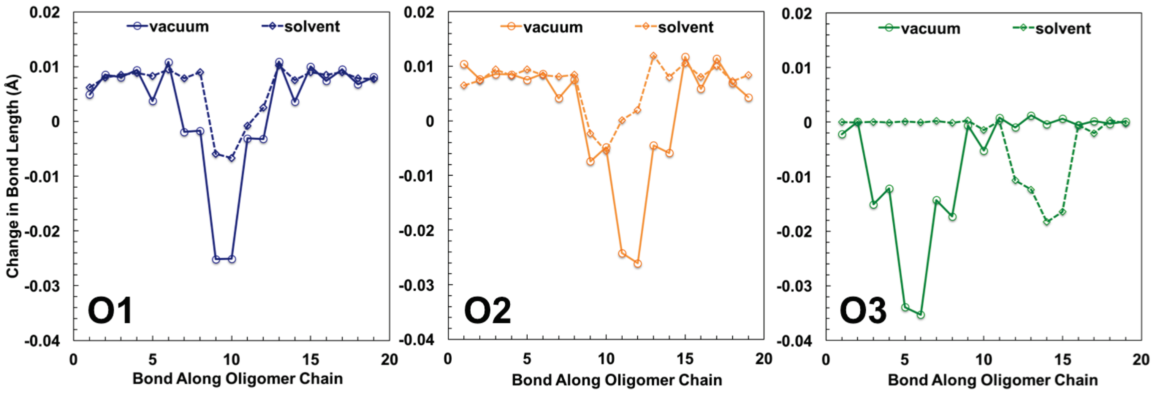

Figure S6. Bond lengths changes for oligomers in vacuum (cation species).

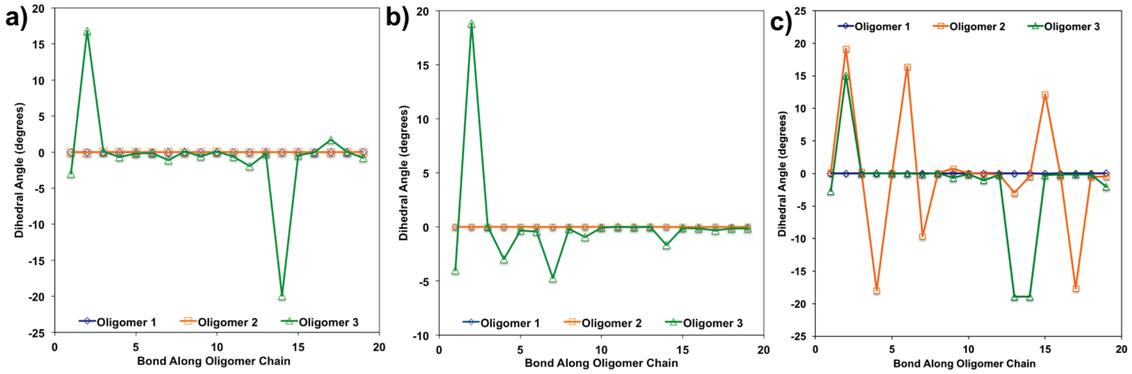

Figure S7. Bond dihedral plots for all oligomers in (a) the neutral geometry, (b) the optimized cation species geometry, and the (c) optimized anion species in vacuum.

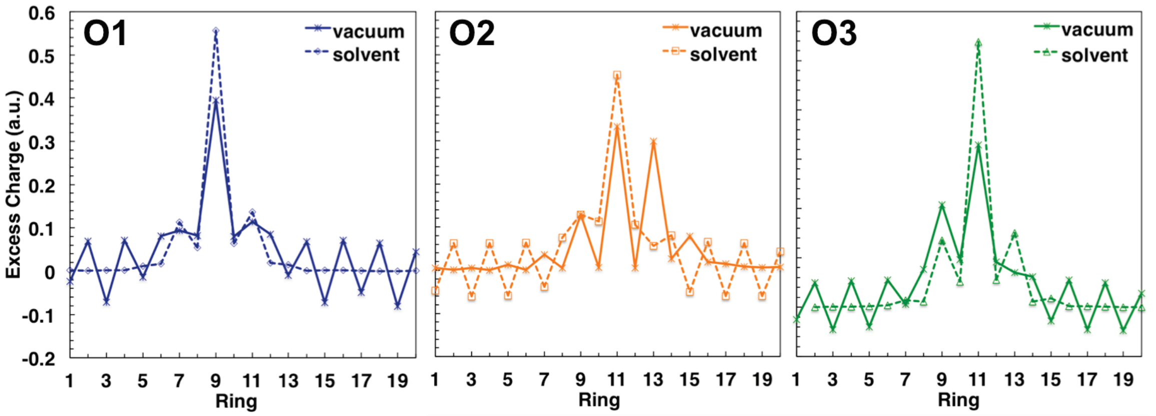

Figure S8. Excess charges of the cation species in vacuum and solvent.

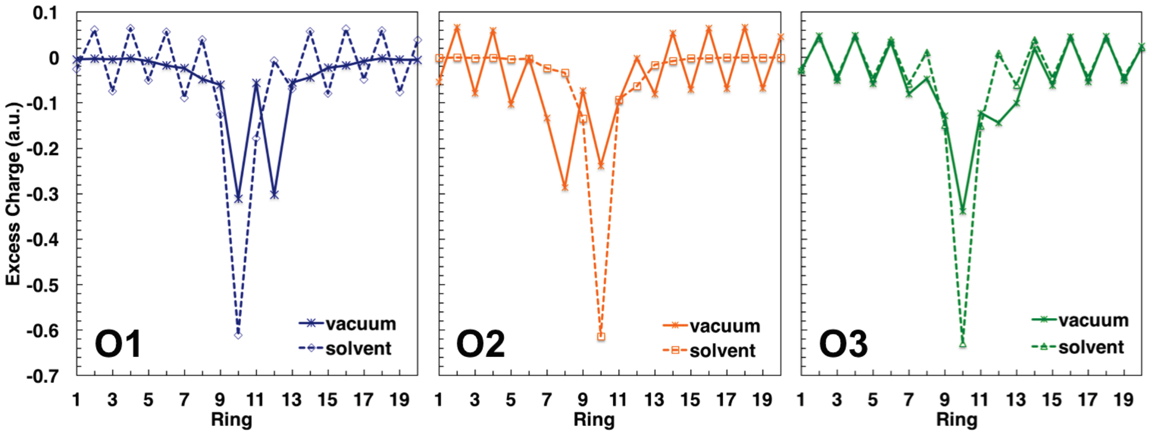

Figure S9. Excess charges of the cation species in vacuum and solvent.

Table S1. Absolute energies of the neutral optimized geometries.

Oligomer	Number of repeat units	Energy (hartrees)
1	1	-3948.182819
	2	-7895.182326
	4	-15789.18618
	5	-19736.1849
2	1	-3948.183182
	2	-7895.182542
	4	-15789.18127
	5	-19736.18062
3	5	-19575.78945

Table S2. Absolute energies of the cation species in neutral optimized geometries.

Oligomer	Number of repeat units	Energy (hartrees)
1	1	-3947.953786
	2	-7894.963822
	4	-15788.96959
	5	-19735.96946
2	1	-3947.957
	2	-7894.965
	4	-15788.96666
	5	-19735.96598
3	5	-19735.96005

Table S3. Absolute energies of the cation optimized geometries.

Oligomer	Number of repeat units	Energy (hartrees)
1	1	-3947.964391

	2	-7894.975136
	4	-15788.97964
	5	-19735.97863
2	1	-3947.965
	2	-7894.975
	4	-15788.97573
	5	-19735.975
3	5	-19735.96968

Table S4. Absolute energies of the neutral species in cation optimized geometries.

Oligomer	Number of repeat units	Energy (hartrees)
1	1	-3948.17272
	2	-7895.171445
	4	-15789.17518
	5	-19736.174
2	1	-3948.173615
	2	-7895.171585
	4	-15789.17016
	5	-19736.169
3	5	-19736.16348

Table S5. Absolute energies of the anion species in neutral optimized geometries.

Oligomer	Number of repeat units	Energy (hartrees)
1	1	-3948.25018
	2	-7895.255
	4	-15789.2523
	5	-19736.26187
2	1	-3948.249
	2	-7895.256
	4	-15789.2484
	5	-19736.24161
3	5	-19736.25301

Table S6. Absolute energies of the anion optimized geometries.

Oligomer	Number of repeat units	Energy (hartrees)
1	1	-3948.25401
	2	-7895.262
	4	-15789.25263
	5	-19736.26728
2	1	-3948.253
	2	-7895.263

	4	-15789.26351
	5	-19736.26346
3	5	-19736.26006

Table S7. Absolute energies of the neutral species in anion optimized geometries.

Oligomer	Number of repeat units	Energy (hartrees)
1	1	-3948.176193
	2	-7895.174265
	4	-15789.17778
	5	-19736.16137
2	1	-3948.176328
	2	-7895.174374
	4	-15789.17242
	5	-19736.17237
3	5	-19736.16603

Table S8. Intramolecular reorganization energies for cation species in vacuum.

**Oligomer**	**n**	**λ_1 (eV)**	**λ_2 (eV)**	**λ (eV)**
1	1	0.286	0.273	0.559
	2	0.305	0.294	0.599
	4	0.271	0.297	0.568
	5	0.248	0.294	0.542
2	1	0.216	0.258	0.474
	2	0.270	0.296	0.566
	4	0.226	0.300	0.545
	5	0.243	0.314	0.557
3	5	0.212	0.271	0.483

Table S9. Intramolecular reorganization energies for cation species in solvent.

**Oligomer**	**n**	**λ_1 (eV)**	**λ_2 (eV)**	**λ (eV)**
1	1	0.243	0.245	0.488
	2	0.264	0.222	0.486
	4	0.340	0.222	0.562
	5	0.330	0.221	0.551
2	1	0.216	0.250	0.466
	2	0.216	0.233	0.449
	4	0.216	0.212	0.428

	5	0.244	0.242	0.486
3	5	0.211	0.223	0.434

Table S10. Intramolecular reorganization energies for anion species in vacuum.

Oligomer	n	λ_1 (eV)	λ_2 (eV)	λ (eV)
1	1	0.103	0.179	0.285
	2	0.189	0.207	0.399
	4	0.009	0.217	0.227
	5	0.146	0.635	0.788
2	1	0.108	0.185	0.295
	2	0.189	0.221	0.413
	4	0.408	0.239	0.652
	5	0.590	0.223	0.819
3	5	0.190	0.240	0.434

Table S11. Intramolecular reorganization energies for anion species in solvent.

Oligomer	n	λ_1 (eV)	λ_2 (eV)	λ (eV)
1	1	0.135	0.480	0.615
	2	0.324	0.149	0.473
	4	0.435	0.214	0.649
	5	0.610	0.635	0.788
2	1	0.162	0.160	0.322
	2	0.162	0.133	0.295
	4	0.135	0.147	0.282
	5	0.590	0.223	0.819
3	5	0.154	0.154	0.308

First principle tight binding modeling of planar polymers

Methodology for images below: Occupied valence molecular orbitals of π-symmetry of oligomers consisting of 16 monomers where localized using combination of top-down and bottom-up approaches [1]. The Fock matrix is then projected onto these LMOs to get tight-binding Hamiltonian. The Hamiltonian of an infinite polymer is obtained by extrapolating the Hamiltonian of the oligomer, taking into account exponential decrease of its elements with spatial separation between LMOs, see Fig. S13.

[1] Z Li, H Li, B Suo, W Liu, Localization of Molecular Orbitals: From Fragments to Molecule, Acc Chem Res 47, 2758 (2014)

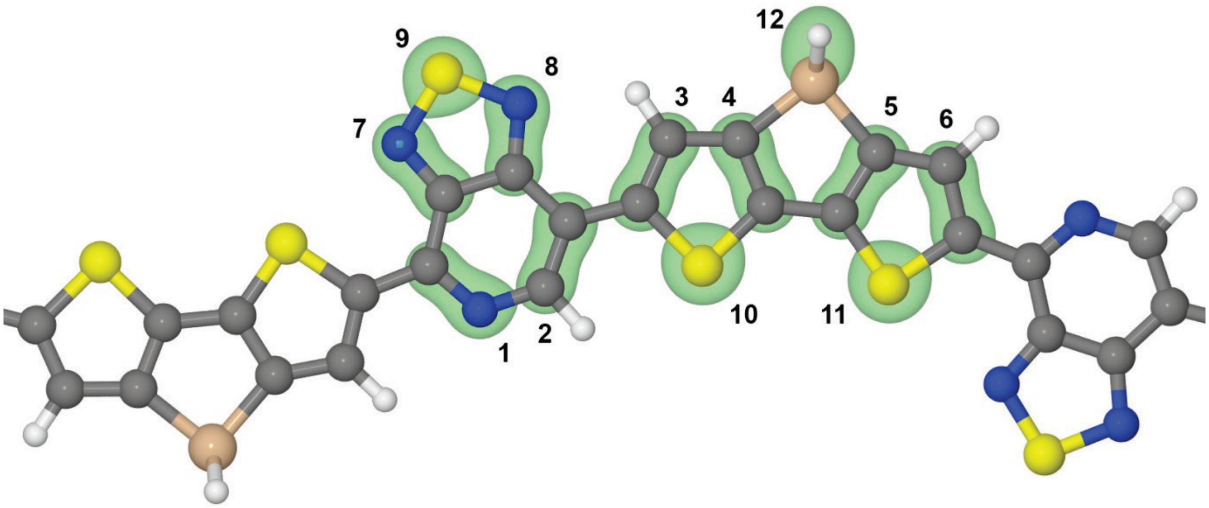

Figure S10. Localized molecular orbitals (LMO): there are 12 LMOs in the repeating unit. Shown here is the part of P1 (O2) polymer.

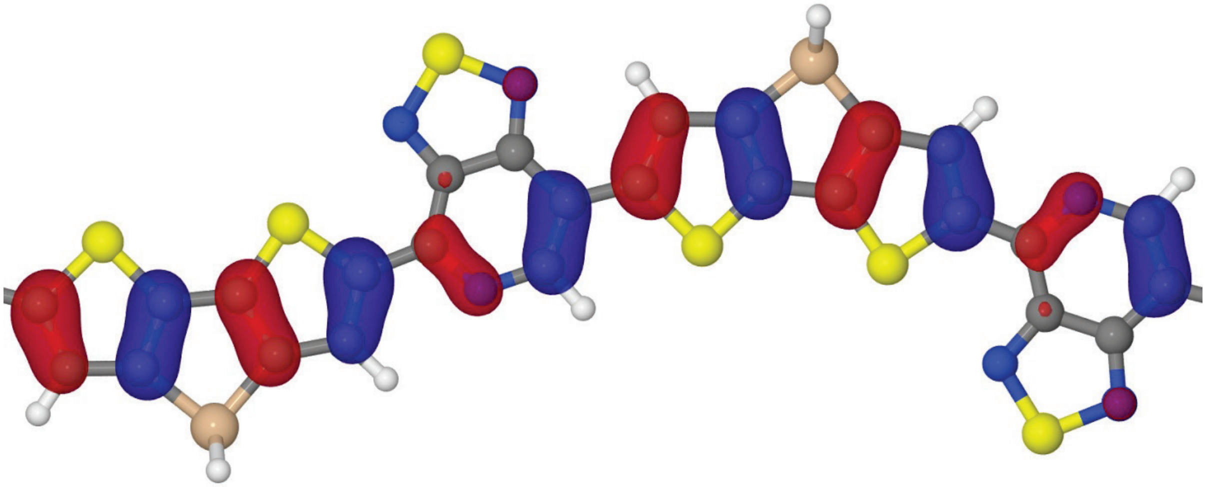

Figure S11. HOMO of the polymer P1 (central part of the long enough oligomer). It is composed primarily of LMOs 1-6 from Fig. S10.

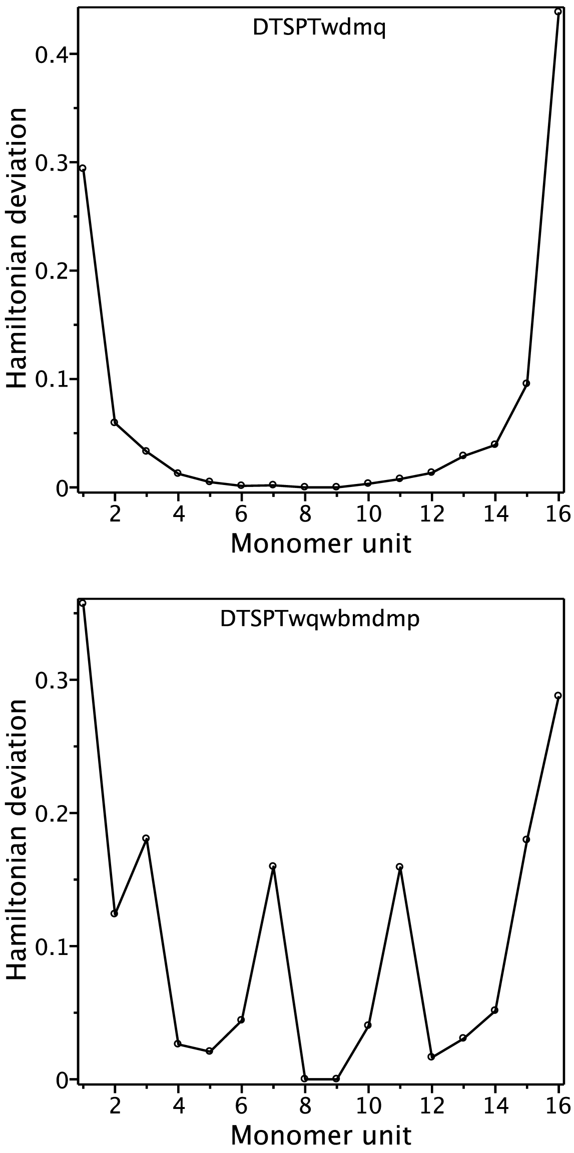

Figure S12. Intra-monomer tight-binding Hamiltonian deviations (Frobenius norm) across the oligomers P1 (left) and P2 (right). The same-order deviations are observed between different polymers (P1,P2,P3) – few tenths of eV, that is not enough to introduce a noticeable disorder in a tight-binding Hamiltonian with 1.2-1.4 eV inter-site couplings.

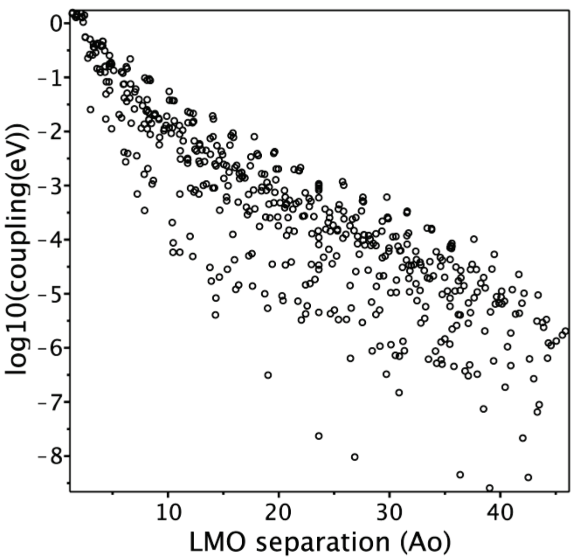

Figure S13. Exponential decrease of elements of tight-binding Hamiltonian with separation between LMOs.

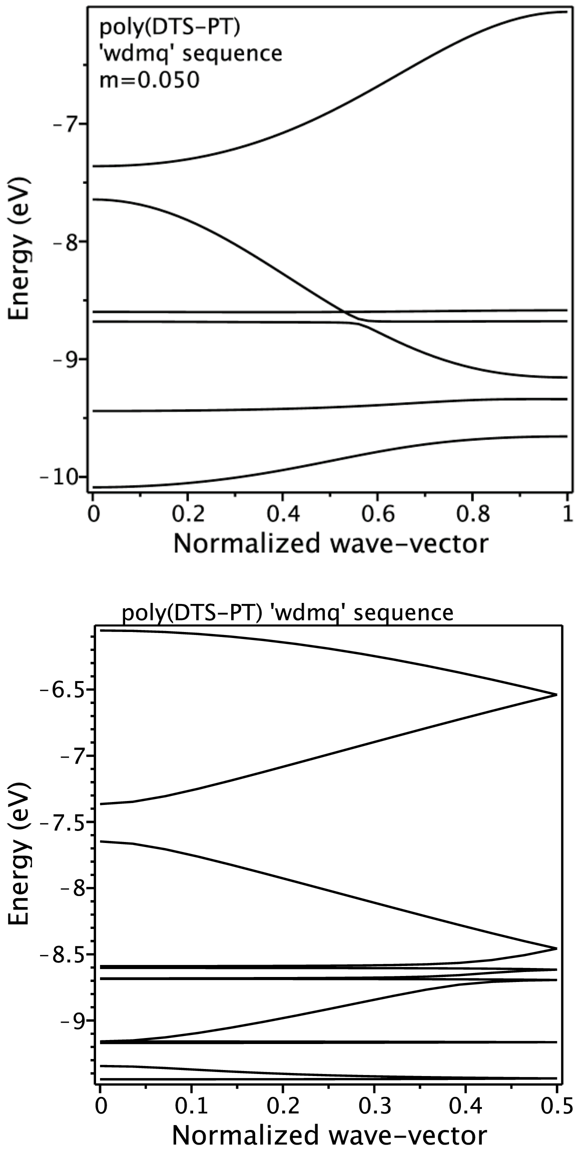

Figure S14. Electronic structure of the valence band of a planar P1 polymer: our simplified approach (π-bands only) vs. straightforward periodic boundary conditions calculations. Both approaches are numerically identical.

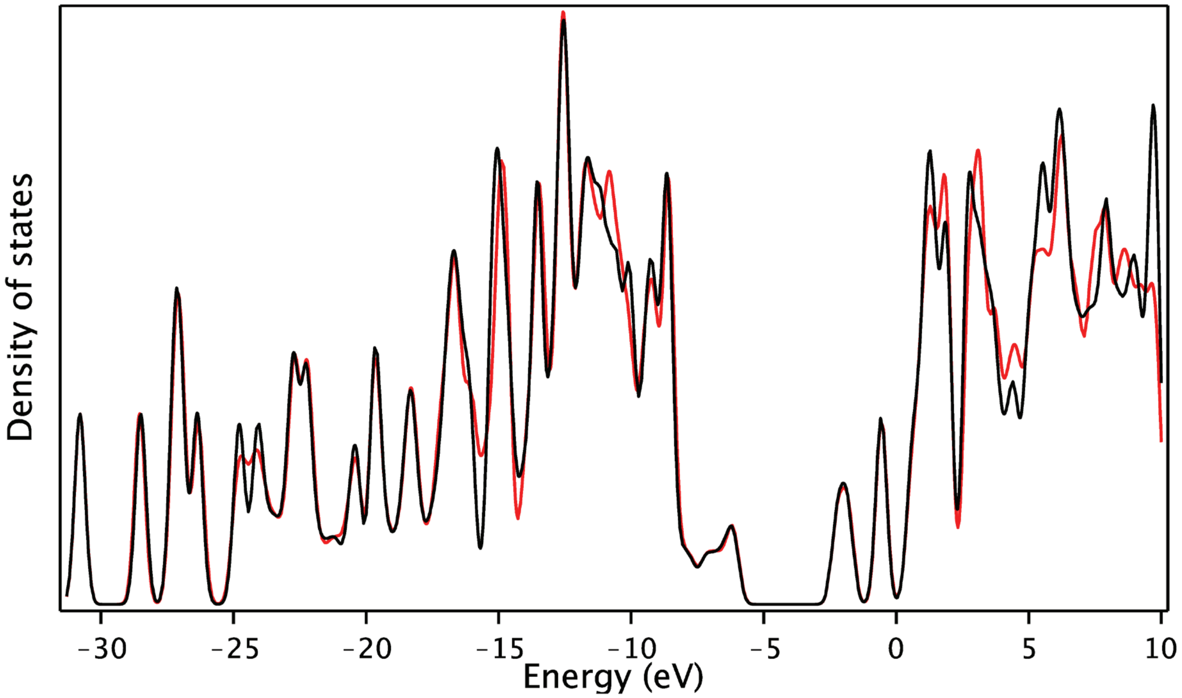

Figure S15. Comparison of electronic density of states: P1 (black) vs. P2 (red).

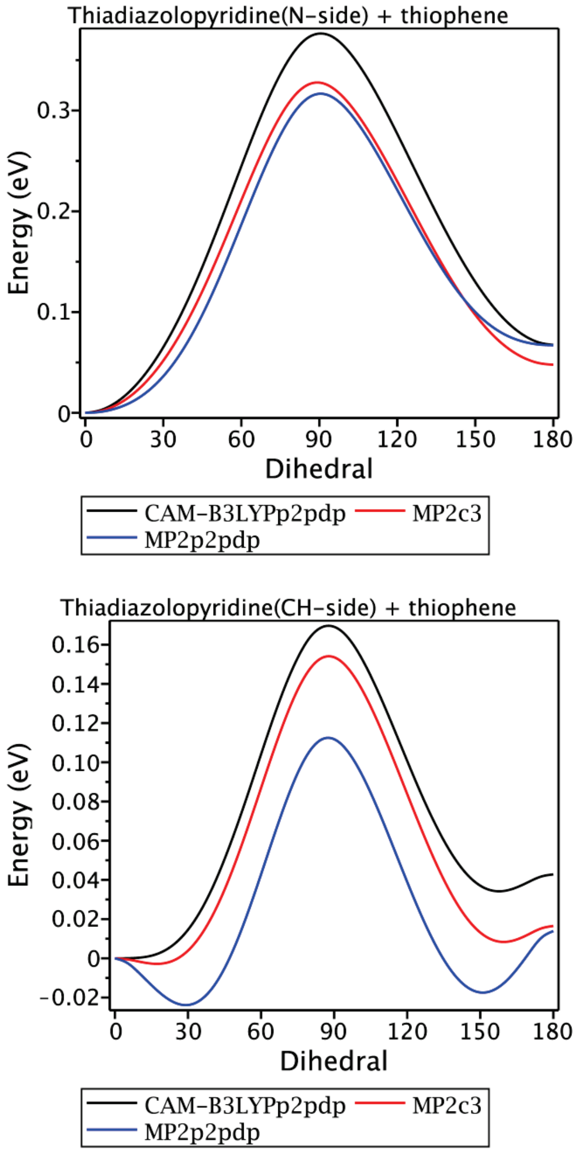

Figure S16. Potential energy surface for the two principal dihedrals determining geometry of the studied polymers. Zero dihedral corresponds to the trans orientation of sulfur atoms.

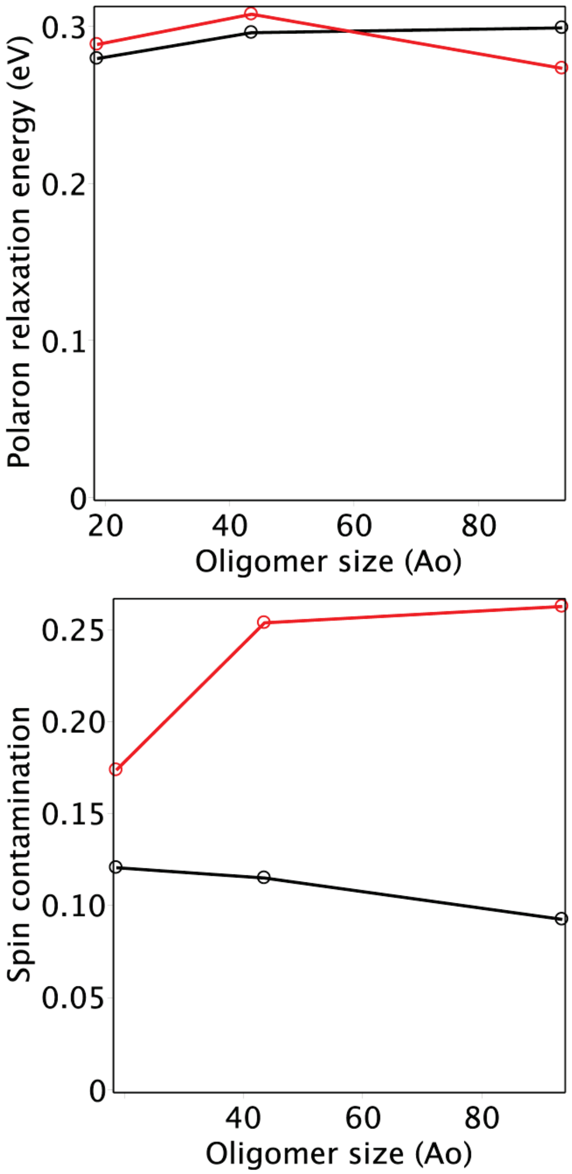

Figure S17. Convergence of polaron relaxation energy (λ_1 and λ_2) and spin contamination with oligomer size.

Tight-binding Hamiltonian for polymer P1 in indices of Fig.S10

i	H[i,i,0](eV)
1	-11.53
2	-10.33
3	-9.85
4	-10.10
5	-10.09

6	-9.84
7	-12.27
8	-11.92
9	-11.06
10	-9.90
11	-9.87
12	-11.39

**i**	**j**	**r**	**H[i,j,r]**	**distance(Ao)**
7	9	0	-1.53	1.5
4	5	0	-1.48	2.2
8	9	0	-1.47	1.4
1	2	0	-1.44	1.8
5	6	0	-1.39	1.8
3	4	0	-1.37	1.8
2	3	0	-1.36	2.5
7	8	0	-1.28	1.7
6	1	1	1.27	2.3
4	10	0	-1.26	1.8
6	11	0	-1.26	1.8
5	11	0	-1.25	1.8
3	10	0	-1.24	1.8
2	8	0	-1.23	2.3
1	7	0	-1.02	2.4
5	12	0	-0.54	2.6
4	12	0	-0.53	2.6
2	10	0	-0.49	3.0
6	2	1	-0.45	4.2
2	4	0	0.45	4.2
1	9	0	0.41	3.7
4	11	0	-0.41	3.3
2	9	0	0.39	3.6
4	6	0	0.39	4.1
5	1	1	-0.37	4.2
3	5	0	0.36	4.1
6	7	1	0.35	3.3
5	10	0	-0.34	3.4
1	3	0	0.30	4.4
11	1	1	0.28	3.5
3	8	0	-0.26	3.0
11	7	1	0.25	3.3
6	9	1	-0.25	4.8
2	7	0	-0.22	2.8
6	3	1	0.21	6.7
3	11	0	0.18	4.9
6	8	1	0.17	4.8

6	10	0	0.17	5.0
11	12	0	0.16	4.4
5	7	1	-0.16	4.8
2	5	0	-0.15	6.3
10	12	0	0.15	4.4
10	11	0	-0.14	3.7
4	1	1	0.14	6.4
3	12	0	-0.14	3.9
11	2	1	-0.13	5.2
3	6	0	-0.12	5.9
3	9	0	0.12	4.4
6	12	0	-0.12	4.0
5	2	1	0.12	6.0
2	6	0	0.09	8.0
6	4	1	-0.09	8.4
3	1	1	-0.09	8.2
5	3	1	-0.08	8.5
3	7	0	-0.08	4.5
1	10	0	0.08	4.7
2	11	0	-0.07	6.7
5	9	1	0.07	6.1
1	4	0	-0.07	6.0
10	1	1	0.06	7.2
4	8	0	0.06	4.8
2	1	1	0.05	10.2
6	10	1	0.05	7.1
5	8	1	-0.05	6.4
11	9	1	-0.05	4.5
4	9	0	-0.04	6.1
4	7	1	0.04	6.6
11	3	1	0.04	7.5
4	2	1	-0.04	8.2
5	4	1	0.04	10.2
6	5	1	0.04	10.4
4	3	1	0.04	10.6
4	7	0	0.03	6.3
4	9	1	-0.03	7.8
5	8	0	-0.03	7.0
1	8	0	-0.02	3.1
3	7	1	-0.02	8.2
1	5	0	0.02	8.1
2	2	1	-0.02	11.9
4	8	1	0.02	8.3
6	6	1	-0.02	12.1
3	3	1	-0.02	12.3
5	10	1	-0.02	8.9

10	2	1	-0.02	8.8
7	10	0	-0.02	5.7
6	8	0	0.02	8.9
2	3	1	0.02	14.1
3	2	1	0.02	10.0
9	10	0	0.02	5.9
3	9	1	0.02	9.2
6	1	2	-0.02	14.2
8	10	0	0.02	4.5
5	9	0	0.02	8.3
11	4	1	-0.02	9.3
1	11	0	0.02	8.4
6	11	1	0.02	10.6
4	4	1	-0.01	12.4
1	1	1	-0.01	11.9
5	5	1	-0.01	12.3
10	7	1	0.01	6.8
5	7	0	-0.01	8.6
8	11	0	-0.01	7.9
3	8	1	-0.01	9.9
10	3	1	0.01	11.1
6	9	0	-0.01	10.2
2	9	1	-0.01	10.5
10	9	1	-0.01	7.7
1	6	0	-0.01	9.7
11	10	1	0.01	8.2
11	8	1	0.01	5.0
2	8	1	0.01	11.4
2	7	1	0.01	9.7

Cartesian Coordinates for n=5 oligomers

Oligomer 1 in vacuum

Atom	X	Y	Z
C	-44.696956709606	-2.581357462190	-0.013726963434
C	-43.696661253613	-3.510346295510	-0.014797236089
C	-42.413902859125	-2.903341895538	-0.013223240763
C	-42.478007460725	-1.530986071638	-0.011005782860
S	-44.096780503628	-0.951529012158	-0.010788723025
H	-45.763603893925	-2.753265717450	-0.014487993224
H	-43.882717253998	-4.577605250131	-0.016627665616
C	-41.135777365807	-0.997539868416	-0.009527743727
C	-40.244302405666	-2.044859893649	-0.010835261997
S	-40.366012190854	0.533427820979	-0.006630053717
C	-38.905148690026	-1.611905417163	-0.009494828465
C	-38.783199973283	-0.236549143532	-0.007182332771
H	-38.052035119272	-2.278674778295	-0.010171400582
C	-40.969404466342	-3.383458853155	-0.013353045466
C	-40.646955782412	-4.196863559560	-1.276127012968
H	-39.588329049896	-4.471745453537	-1.297254995232
H	-41.234078959423	-5.119534201611	-1.299639318931
H	-40.871096709174	-3.624179509894	-2.178647130612

C	-40.648094676523	-4.200978885338	1.247051775791
H	-41.235205713147	-5.123741623762	1.267010452865
H	-39.589478575036	-4.475895956376	1.268256891038
H	-40.873085570846	-3.631256435809	2.151233391515
C	-37.541966983620	0.526392197255	-0.005388056808
C	-36.302602319417	-0.078290839408	-0.006113093142
C	-37.485793276020	1.958438189693	-0.002724544508
H	-36.233110800211	-1.160680963029	-0.008139582141
C	-36.216502880032	2.637115375139	-0.001031642789
C	-35.004398604905	1.858048082095	-0.002043124825
C	-33.678544785335	2.440033872497	-0.000471608884
C	-33.291833173954	3.764792657292	0.002160566684
S	-32.304763009128	1.349516657016	-0.002000823966
C	-31.891707739351	3.905606988495	0.002934487018
H	-34.010472256080	4.572894050544	0.003411087932
C	-31.235970659008	2.691012993167	0.000894073078
C	-29.816446545295	2.930064679493	0.001857020058
C	-29.595085272209	4.292721679238	0.004491750712
S	-28.367149384884	2.013263444210	0.000659400023
C	-28.226711063771	4.618662669879	0.005583889511
C	-27.426318999596	3.493970674933	0.003750556653
H	-27.813443339822	5.617843466259	0.007597237184
C	-30.906112668079	5.064362912774	0.005475372058
C	-25.983485322086	3.377544961229	0.004027919591
C	-25.092628330505	4.512043921514	0.006425603627
C	-24.123237327774	1.977720597669	0.002058129047
C	-23.667860537719	4.294296308227	0.006503691239
C	-23.150636156124	2.953803377433	0.004198510827
H	-23.831074865868	0.931306085976	0.000205535175
N	-38.519779784403	2.793742684570	-0.001546986797
N	-36.326668935069	3.961242057663	0.001368453001
N	-25.431992056003	5.795850270553	0.008684925111
N	-22.974516652826	5.429782803308	0.008838531990
N	-25.463642309738	2.171216457007	0.001968200744
N	-35.101423394914	0.547419074466	-0.004526059627
S	-37.916101522128	4.303841304493	0.001444787868
S	-24.050096892838	6.650994955358	0.010736864864
C	-31.052142174881	5.927051148097	1.268554338628
H	-32.031077308619	6.414447734538	1.289285051082
H	-30.951002383869	5.321063572342	2.171546832390
H	-30.286142579944	6.707496293750	1.290664563220
C	-31.051220849860	5.931831607232	-1.254431496024
H	-32.030127233190	6.419332790203	-1.274015331873
H	-30.285182448840	6.712328899764	-1.273030832305
H	-30.949449429234	5.329268922553	-2.159642226964
C	-21.727497146373	2.653341175990	0.004127857299
C	-20.663080193210	3.528496053870	0.005912848537
S	-21.172676447118	0.980735896425	0.001497194438
C	-19.416853554076	2.867539824411	0.005159296245
H	-20.806199766370	4.599729461416	0.007676795249
C	-19.535890474058	1.497522186206	0.002823052791
C	-17.954273931347	3.288270939051	0.006281946111
C	-18.220853615534	0.909746696162	0.002203643259
C	-17.286012776317	1.920855143416	0.004166915931
C	-17.599967854048	4.087685772117	1.269491052461
C	-17.599251193144	4.091931664227	-1.254029045244
S	-17.514046442711	-0.652214083101	-0.000183039099
C	-15.966970295945	1.433304425875	0.003728908447
H	-16.531192577748	4.319798564901	1.290617257764
H	-18.150376519903	5.032516002133	1.292321085603
H	-17.847066944724	3.524506932591	2.172013506552
H	-18.149624735356	5.036847952063	-1.273982461552
H	-16.530459215225	4.324088876771	-1.273780514411
H	-17.845860855435	3.531804866814	-2.158582478752
C	-15.902135493768	0.053369279749	0.001419548521
H	-15.087157154090	2.064250871565	0.005061032885
C	-14.693196315795	-0.759314889502	0.000329308031
C	-13.430451448106	-0.204782498657	0.001216493781
C	-14.695243887732	-2.192323078537	-0.001744363081
H	-13.317491631409	0.873959125256	0.002732962294

N	-12.255754481416	-0.878054943882	0.000367721209
C	-13.454432658452	-2.921518788094	-0.002685177516
N	-15.762118220789	-2.985009394916	-0.002997249405
C	-12.211729499004	-2.191928073586	-0.001511076856
N	-13.618019780458	-4.240049305737	-0.004613599412
S	-15.219891103784	-4.518439863881	-0.005167459626
C	-10.910703050762	-2.826133089071	-0.002295818046
C	-10.576563170477	-4.165412794116	-0.004174991875
S	-9.494816834671	-1.790763023551	-0.000674567514
C	-9.183323301092	-4.361418362542	-0.004339534124
H	-11.326603593008	-4.944488764710	-0.005353870891
C	-8.480235062320	-3.173355636618	-0.002584001240
C	-8.243712429537	-5.557883175886	-0.005978531706
C	-7.071338468613	-3.467776152121	-0.002893683073
C	-6.903474053984	-4.838020402376	-0.004840455261
C	-8.422855106456	-6.414768472861	-1.268746282970
C	-8.423053248687	-6.418391226628	1.254295477145
S	-5.587466091736	-2.608095178012	-0.001529885013
C	-5.548713614030	-5.217081438976	-0.005276782261
H	-9.420017830048	-6.863677413588	-1.289560277236
H	-8.297891976720	-5.813626739154	-2.171994997029
H	-7.687860001305	-7.224482241887	-1.290259308252
H	-9.420215121796	-6.867366839096	1.273659287652
H	-7.688053588407	-7.228156215949	1.273601151587
H	-8.298241309436	-5.819844931835	2.159287039174
C	-4.705298772195	-4.124350019798	-0.003657946867
H	-5.174519867424	-6.231556120440	-0.006700598502
C	-3.258871843448	-4.063634331524	-0.003472749088
C	-2.412155781317	-5.231272679176	-0.005076582560
N	-2.693404612139	-2.878073806884	-0.001743111932
C	-0.980125006439	-5.068118549136	-0.004745287187
N	-2.800369154248	-6.501202451234	-0.006910255384
C	-1.346639579699	-2.735791797129	-0.001447260728
C	-0.412034262237	-3.748360043821	-0.002828975406
N	-0.330678071536	-6.229286859706	-0.006350917654
S	-1.452137115098	-7.408454674864	-0.008100716288
H	-1.014790102585	-1.701298785406	0.000058468925
C	1.021413795081	-3.502114039141	-0.002382793935
C	2.052139831863	-4.416764469339	-0.003529444123
S	1.638809874148	-1.851585179849	-0.000097945870
C	3.322380540401	-3.803194682400	-0.002599500433
H	1.868753148078	-5.481856090940	-0.004981072350
C	3.254868618813	-2.429654888992	-0.000755132791
C	4.768234970905	-4.278398049266	-0.003124025549
C	4.590936524677	-1.891560247284	0.000075637023
C	5.487276022663	-2.936925582288	-0.001260471329
C	5.092762708351	-5.090932004544	-1.265964028867
C	5.092563802449	-5.094329355446	1.257575291209
S	5.355517690954	-0.357095838135	0.002199958605
C	6.823646558753	-2.498960754280	-0.000575295474
H	6.152113715597	-5.362856941824	-1.286673874913
H	4.507431412688	-6.014536769010	-1.288681572575
H	4.867189045646	-4.519210572966	-2.168751984905
H	4.507235413598	-6.017996053217	1.277711952536
H	6.151913215820	-5.366302788288	1.277723711551
H	4.866841288138	-4.525043272923	2.161863788088
C	6.939933826061	-1.122394050161	0.001271456874
H	7.679314095044	-3.162284582321	-0.001413688625
C	8.178233309951	-0.355132331134	0.002394601665
C	9.419581261157	-0.956004785042	0.001799913123
C	8.229180828619	1.076974880214	0.004203600317
H	9.492545750656	-2.038195864968	0.000466311669
N	10.618409393151	-0.326663904018	0.002733832344
C	9.496106100914	1.759755736524	0.005216169940
N	7.192363450392	1.908586729713	0.005125132007
C	10.710998169450	0.984693306364	0.004388470694
N	9.381401837914	3.083430700768	0.006868488630
S	7.790956028891	3.420920300832	0.007109696201
C	12.034488630273	1.570508196798	0.005282005473
C	12.417592295099	2.896630412605	0.006989324225

S	13.411441308213	0.483879842973	0.004108824463
C	13.817044838546	3.041373261438	0.007325950708
H	11.696681272895	3.702731467565	0.007911601179
C	14.476093078549	1.828306966894	0.005887026734
C	14.799875272225	4.202570212646	0.008879917891
C	15.894855401445	2.070884340863	0.006343973423
C	16.112794575741	3.434052659538	0.008063474901
C	14.652137538596	5.065714567946	1.271457187521
C	14.652411418307	5.068860400413	-1.251572825846
S	17.346271294608	1.157452254796	0.005377994170
C	17.480527603615	3.763269428008	0.008616564655
H	13.672139633898	5.550961883032	1.292043524873
H	14.754832905293	4.460585615334	2.174848856063
H	15.416380014823	5.847881091243	1.292877039242
H	13.672419731825	5.554161562663	-1.271159519088
H	15.416661448856	5.851074442864	-1.270876942788
H	14.755299156781	4.465986040247	-2.156448810335
C	18.283356019594	2.640394505497	0.007298440710
H	17.891581596604	4.763374224031	0.009901872856
C	19.726603251613	2.526763212335	0.007301003108
C	20.615524312231	3.662592484624	0.008785598616
N	20.248236431461	1.321283229509	0.005872637860
C	22.040613712462	3.447051560756	0.008663770070
N	20.274124072204	4.945902227456	0.010327768237
C	21.588881465562	1.129725109396	0.005772577016
C	22.559962200690	2.107371438482	0.007072129900
N	22.732187511756	4.583632289401	0.010130149083
S	21.654720139420	5.803113145403	0.011527552096
H	21.882583211068	0.083762259653	0.004517724150
C	23.983422948138	1.808766721566	0.006856157602
C	25.047023181672	2.684989701083	0.008004181281
S	24.539818715908	0.136705144951	0.004937216362
C	26.293897410607	2.025221726561	0.007335863297
H	24.902843805638	3.756096452451	0.009267089255
C	26.175980307030	0.655083270320	0.005694741300
C	27.756266031577	2.446956371373	0.007972832356
C	27.491342342709	0.068263057574	0.005115515923
C	28.425528830230	1.079949552673	0.006405588549
C	28.110277225492	3.247226390236	1.270724081948
C	28.110508978670	3.250232152328	-1.252803011548
S	28.198848875031	-1.493298533463	0.003348322863
C	29.744882959419	0.593054140947	0.005953074138
H	29.178906008774	3.480022838050	1.291534641638
H	27.559291530580	4.191728726012	1.293178386380
H	27.863707687970	2.684350582674	2.173580085246
H	27.559524022450	4.194783534110	-1.273110371352
H	29.179140712664	3.483081055280	-1.272860112324
H	27.864109623226	2.689507394279	-2.157042938684
C	29.810374243300	-0.786867676797	0.004313626338
H	30.624429134417	1.224381878031	0.006795595986
C	31.019479336128	-1.599354485388	0.003438914072
C	32.282260849487	-1.044926201101	0.004064570266
C	31.017278627984	-3.032361583119	0.001872788422
H	32.395293547264	0.033818422577	0.005231238403
N	33.456942478175	-1.718322974997	0.003364613515
C	32.257970530049	-3.761676628865	0.001107740776
N	29.950310699320	-3.824918866852	0.000979221975
C	33.500789179964	-3.032205251284	0.001936772046
N	32.094196085551	-5.080184666185	-0.000334558968
S	30.492321884189	-5.358413514763	-0.000674031875
C	34.801521934988	-3.666979548152	0.001286601673
C	35.134513524905	-5.006543018202	-0.000134822565
S	36.218353052839	-2.632881589938	0.002435481462
C	36.527506099874	-5.203778971170	-0.000314922423
H	34.383842097307	-5.784992466166	-0.000983257396
C	37.231780446415	-4.016396796641	0.000967875761
C	37.465727948143	-6.401240413255	-0.001574650509
C	38.640422863544	-4.312368812762	0.000678425839
C	38.806661823451	-5.682830415941	-0.000782860263
C	37.285567845231	-7.258387517498	-1.264022677697

C	37.285519195139	-7.261080678136	1.259034344264
S	40.125485530429	-3.454616538201	0.001625577944
C	40.160864770833	-6.063619456946	-0.001172100027
H	36.287914040868	-7.706211563532	-1.284602220151
H	37.411125501732	-6.657714710420	-2.167500949439
H	38.019671532781	-8.068914375135	-1.285290622336
H	36.287864293814	-7.708946935653	1.278620743206
H	38.019621446566	-8.071651697928	1.278600384822
H	37.411043540238	-6.662337167144	2.163796858205
C	41.005719443284	-4.972009317072	-0.000001156899
H	40.533685502853	-7.078585654163	-0.002251462342
C	42.452244388466	-4.913797929031	0.000066517714
C	43.296583589373	-6.083199364029	-0.001167047978
N	43.020112821310	-3.729385673055	0.001325462456
C	44.728910452833	-5.923012904589	-0.000995321707
N	42.905777048469	-7.352337401712	-0.002509622354
C	44.367217082778	-3.589906993211	0.001476167735
C	45.299707155960	-4.604411972629	0.000398971494
N	45.375954082309	-7.085528614485	-0.002221092962
S	44.252088721138	-8.262381774326	-0.003458136483
H	44.701239989798	-2.556107650813	0.002578803342
C	46.733742527164	-4.361466420318	0.000653978578
C	47.762041141443	-5.278810884643	-0.000264105529
S	47.355482808163	-2.712562376511	0.002332174323
C	49.033851512864	-4.668604496091	0.000360894276
H	47.575896693921	-6.343409625084	-0.001342478878
C	48.970088972473	-3.294888473387	0.001748259214
C	50.478255352774	-5.147966578001	-0.000119080418
C	50.307763309088	-2.760584616691	0.002290777585
C	51.201056071638	-3.808593063269	0.001233823490
C	50.800345523119	-5.961845106589	-1.262721381988
C	50.800337649277	-5.964395356830	1.260837885876
S	51.077028967137	-1.228412811504	0.003832679500
C	52.538666437688	-3.374671603265	0.001661154812
H	51.858903126459	-6.236847473942	-1.283416062941
H	50.212350451198	-6.883761107840	-1.285108405378
H	50.576361493861	-5.389771374740	-2.165681674547
H	50.212328441704	-6.886345422304	1.281364816263
H	51.858891366632	-6.239454051109	1.280975227329
H	50.576363635823	-5.394140902891	2.164950633699
C	52.659118911664	-1.998478867191	0.003035966419
H	53.392269438482	-4.040640042534	0.000980728268
C	53.899973053597	-1.235371259474	0.003781875947
C	55.139135731560	-1.840649815298	0.003204366260
C	53.956027646499	0.196563390629	0.005162715592
H	55.208367930928	-2.923065998738	0.002173680349
N	56.340174508012	-1.215432810215	0.003798480352
C	55.225369642306	0.874807797523	0.005810052373
N	52.922180229404	1.031891632595	0.005974453369
C	56.437364875055	0.095452500377	0.005050277565
N	55.115413307915	2.198930335075	0.007088846681
S	53.526223725129	2.542016358695	0.007429794552
C	57.763392936937	0.676284527726	0.005584215996
C	58.151918941694	2.000608643239	0.006831443349
S	59.135533745763	-0.416231148097	0.004523827457
C	59.552357601076	2.139370612541	0.006931344692
H	57.434446678096	2.809780604127	0.007612098876
C	60.205751837846	0.923652381314	0.005766825706
C	60.540230379307	3.296367062198	0.007995088845
C	61.626087034792	1.160084263230	0.005953178031
C	61.849764005179	2.522041112152	0.007233165261
C	60.396312436090	4.160588942163	1.270306111403
C	60.396243857349	4.162954059292	-1.252685515749
S	63.072755370638	0.239794647016	0.005044695409
C	63.219672961163	2.844410195554	0.007501513261
H	59.418421737433	4.650067683797	1.290804605836
H	60.496520462955	3.555391600075	2.173934366625
H	61.163886114256	4.939472641662	1.291290062897
H	59.418345717176	4.652457515845	-1.272219672272
H	61.163805875046	4.941886847438	-1.272246884495

H	60.496415892067	3.559453317139	-2.157451760066
C	64.015966362783	1.717929006919	0.006419632643
H	63.636440052666	3.842305224718	0.008433752625
C	65.461364899221	1.598147120851	0.006266751725
C	66.346585099937	2.741954397582	0.007319846979
N	65.971692493205	0.389169601564	0.005116239025
C	67.766774854274	2.517378522879	0.007065326974
N	66.014163224541	4.028381241018	0.008542989969
C	67.323222109046	0.195817678885	0.004896434340
C	68.258780055437	1.185291235539	0.005798004801
N	68.477664753221	3.643036095178	0.008103199580
S	67.407750254143	4.869683825710	0.009291627341
H	67.628701254219	-0.846097731394	0.003909138325
H	69.321669597475	0.982030741554	0.005575242909

Oligomer 1 in solvent

Atom	X	Y	Z
C	56.423331000000	3.156427000000	-0.194155000000
C	55.383702000000	4.054382000000	-0.191911000000
C	54.120418000000	3.407145000000	-0.157389000000
C	54.226601000000	2.025067000000	-0.134252000000
S	55.876294000000	1.494446000000	-0.154203000000
H	57.484807000000	3.360248000000	-0.216495000000
H	55.534968000000	5.127666000000	-0.213909000000
C	52.908390000000	1.450569000000	-0.101069000000
C	51.970485000000	2.475131000000	-0.102607000000
S	52.184011000000	-0.115967000000	-0.052209000000
C	50.648118000000	2.001730000000	-0.066251000000
C	50.559659000000	0.610721000000	-0.037414000000
H	49.775799000000	2.644060000000	-0.057075000000
C	52.655149000000	3.841968000000	-0.138462000000
C	52.280962000000	4.631994000000	-1.412383000000
H	51.212941000000	4.872321000000	-1.413744000000
H	52.839520000000	5.572398000000	-1.456411000000
H	52.505498000000	4.055356000000	-2.313889000000
C	52.326578000000	4.673642000000	1.121573000000
H	52.883056000000	5.616308000000	1.113342000000
H	51.258581000000	4.911621000000	1.155213000000
H	52.586809000000	4.128104000000	2.032793000000
C	49.351100000000	-0.188378000000	-0.003992000000
C	48.078311000000	0.387796000000	-0.043351000000
C	49.331409000000	-1.622787000000	0.073560000000
H	47.985736000000	1.467413000000	-0.110197000000
C	48.072116000000	-2.339125000000	0.110510000000
C	46.836324000000	-1.595055000000	0.069001000000
C	45.529841000000	-2.202308000000	0.105751000000
C	45.156811000000	-3.542132000000	0.184507000000
S	44.118535000000	-1.129888000000	0.047107000000
C	43.762597000000	-3.710895000000	0.197964000000
H	45.888870000000	-4.337557000000	0.228584000000
C	43.069281000000	-2.502308000000	0.129616000000
C	41.664839000000	-2.769012000000	0.149109000000
C	41.464846000000	-4.147715000000	0.229517000000
S	40.184158000000	-1.877587000000	0.099605000000
C	40.106760000000	-4.502031000000	0.251624000000
C	39.265568000000	-3.392721000000	0.188244000000
H	39.719024000000	-5.510287000000	0.310266000000
C	42.797678000000	-4.892859000000	0.270511000000
C	37.828076000000	-3.306071000000	0.183515000000
C	36.951129000000	-4.452604000000	0.246502000000
C	35.945005000000	-1.921894000000	0.104552000000
C	35.513526000000	-4.255846000000	0.233327000000
C	34.972569000000	-2.922485000000	0.158530000000
H	35.631125000000	-0.882151000000	0.046474000000
N	50.396170000000	-2.429639000000	0.119140000000
N	48.214338000000	-3.665237000000	0.183308000000
N	37.315474000000	-5.734768000000	0.318714000000
N	34.829268000000	-5.403142000000	0.296480000000
N	37.281082000000	-2.092229000000	0.115540000000

N	46.900070000000	-0.265503000000	-0.007503000000
S	49.828488000000	-3.970394000000	0.202203000000
S	35.932945000000	-6.620084000000	0.366342000000
C	42.966537000000	-5.683004000000	1.587830000000
H	43.957617000000	-6.145433000000	1.630290000000
H	42.853128000000	-5.029785000000	2.457287000000
H	42.217771000000	-6.478573000000	1.653632000000
C	42.958903000000	-5.835977000000	-0.942934000000
H	43.948020000000	-6.304322000000	-0.932894000000
H	42.206808000000	-6.630328000000	-0.909521000000
H	42.844247000000	-5.291864000000	-1.884371000000
C	33.554308000000	-2.638028000000	0.140831000000
C	32.481518000000	-3.524796000000	0.182424000000
S	32.975074000000	-0.954882000000	0.054830000000
C	31.231607000000	-2.880837000000	0.146063000000
H	32.633283000000	-4.594149000000	0.236700000000
C	31.333139000000	-1.495465000000	0.076909000000
C	29.766781000000	-3.317298000000	0.163158000000
C	30.021118000000	-0.925662000000	0.044323000000
C	29.081957000000	-1.952514000000	0.092685000000
C	29.413168000000	-4.064953000000	1.468227000000
C	29.420013000000	-4.189756000000	-1.064082000000
S	29.294185000000	0.639584000000	-0.039597000000
C	27.761186000000	-1.481311000000	0.062467000000
H	28.345880000000	-4.306662000000	1.492558000000
H	29.974739000000	-5.002051000000	1.533980000000
H	29.649725000000	-3.458631000000	2.346828000000
H	29.975473000000	-5.132274000000	-1.031303000000
H	28.351356000000	-4.426364000000	-1.074267000000
H	29.668138000000	-3.675681000000	-1.996660000000
C	27.672263000000	-0.090041000000	-0.009372000000
H	26.889039000000	-2.123035000000	0.090443000000
C	26.464068000000	0.706855000000	-0.056835000000
C	25.190990000000	0.129056000000	-0.028732000000
C	26.444823000000	2.141013000000	-0.138317000000
H	25.098961000000	-0.950911000000	0.033227000000
N	24.013291000000	0.781024000000	-0.072035000000
C	25.185668000000	2.856358000000	-0.185198000000
N	27.509461000000	2.947698000000	-0.179523000000
C	23.949664000000	2.111531000000	-0.149529000000
N	25.327749000000	4.182103000000	-0.261021000000
S	26.941785000000	4.488498000000	-0.271036000000
C	22.644571000000	2.718027000000	-0.194663000000
C	22.272701000000	4.058787000000	-0.274794000000
S	21.231841000000	1.646529000000	-0.146871000000
C	20.879576000000	4.228421000000	-0.297884000000
H	23.005618000000	4.853708000000	-0.313051000000
C	20.184546000000	3.019736000000	-0.235687000000
C	19.916204000000	5.411117000000	-0.376156000000
C	18.780786000000	3.287658000000	-0.265183000000
C	18.582839000000	4.666785000000	-0.345528000000
C	20.095047000000	6.202414000000	-1.691476000000
C	20.069182000000	6.352856000000	0.839455000000
S	17.298512000000	2.397895000000	-0.227755000000
C	17.225462000000	5.022723000000	-0.377157000000
H	21.086728000000	6.664150000000	-1.726335000000
H	19.987398000000	5.550158000000	-2.562379000000
H	19.347335000000	6.998566000000	-1.761748000000
H	21.058450000000	6.820935000000	0.836865000000
H	19.317549000000	7.147412000000	0.801496000000
H	19.947714000000	5.807771000000	1.779465000000
C	16.382733000000	3.914217000000	-0.321326000000
H	16.839293000000	6.031450000000	-0.437433000000
C	14.944886000000	3.830320000000	-0.327529000000
C	14.071167000000	4.978920000000	-0.394457000000
N	14.394876000000	2.617554000000	-0.266279000000
C	12.633162000000	4.785383000000	-0.392319000000
N	14.438869000000	6.260496000000	-0.461130000000
C	13.058428000000	2.449985000000	-0.265530000000
C	12.088610000000	3.453025000000	-0.324493000000

N	11.951979000000	5.934367000000	-0.458044000000
S	13.058742000000	7.148905000000	-0.517063000000
H	12.741798000000	1.410859000000	-0.211861000000
C	10.669719000000	3.172246000000	-0.317732000000
C	9.599913000000	4.062628000000	-0.361733000000
S	10.084604000000	1.490493000000	-0.244043000000
C	8.347959000000	3.422608000000	-0.336522000000
H	9.755322000000	5.131738000000	-0.409716000000
C	8.444527000000	2.036445000000	-0.273720000000
C	6.884896000000	3.864202000000	-0.360477000000
C	7.130342000000	1.471002000000	-0.251759000000
C	6.195168000000	2.501503000000	-0.300690000000
C	6.541750000000	4.618960000000	-1.664258000000
C	6.533509000000	4.732042000000	0.868723000000
S	6.396857000000	-0.092060000000	-0.182149000000
C	4.872547000000	2.035202000000	-0.281726000000
H	5.475354000000	4.863908000000	-1.694223000000
H	7.106453000000	5.554689000000	-1.722132000000
H	6.782094000000	4.016000000000	-2.544140000000
H	7.092324000000	5.672809000000	0.843846000000
H	5.465608000000	4.972208000000	0.873485000000
H	6.774136000000	4.212702000000	1.800346000000
C	4.777977000000	0.643950000000	-0.217749000000
H	4.003137000000	2.680442000000	-0.313186000000
C	3.566238000000	-0.148090000000	-0.179540000000
C	2.296033000000	0.436405000000	-0.194556000000
C	3.540109000000	-1.583202000000	-0.120838000000
H	2.209209000000	1.517745000000	-0.236481000000
N	1.115238000000	-0.210318000000	-0.160224000000
C	2.277463000000	-2.293009000000	-0.083641000000
N	4.600825000000	-2.395629000000	-0.093440000000
C	1.045105000000	-1.541641000000	-0.105433000000
N	2.413048000000	-3.620455000000	-0.028761000000
S	4.025570000000	-3.934880000000	-0.025276000000
C	-0.263122000000	-2.141770000000	-0.068201000000
C	-0.642877000000	-3.481728000000	-0.015378000000
S	-1.669409000000	-1.060954000000	-0.087587000000
C	-2.036953000000	-3.643375000000	0.009593000000
H	0.085316000000	-4.281708000000	0.003193000000
C	-2.724562000000	-2.429255000000	-0.023381000000
C	-3.007762000000	-4.821276000000	0.066308000000
C	-4.129789000000	-2.688673000000	0.007478000000
C	-4.336413000000	-4.067881000000	0.058596000000
C	-2.828477000000	-5.642852000000	1.362881000000
C	-2.866452000000	-5.736732000000	-1.170638000000
S	-5.606043000000	-1.788443000000	-0.000259000000
C	-5.696019000000	-4.415457000000	0.090761000000
H	-1.840244000000	-6.112789000000	1.382539000000
H	-2.926907000000	-5.009326000000	2.248583000000
H	-3.582006000000	-6.434602000000	1.419259000000
H	-1.880253000000	-6.211082000000	-1.183089000000
H	-3.623072000000	-6.527107000000	-1.146939000000
H	-2.988637000000	-5.170056000000	-2.097701000000
C	-6.531444000000	-3.300333000000	0.065293000000
H	-6.088660000000	-5.422681000000	0.131283000000
C	-7.968544000000	-3.206184000000	0.085469000000
C	-8.850645000000	-4.349423000000	0.129184000000
N	-8.509454000000	-1.988058000000	0.061614000000
C	-10.287022000000	-4.144740000000	0.147787000000
N	-8.492552000000	-5.635164000000	0.156025000000
C	-9.844432000000	-1.810174000000	0.079152000000
C	-10.821517000000	-2.806864000000	0.123328000000
N	-10.976714000000	-5.289767000000	0.188951000000
S	-9.879189000000	-6.514020000000	0.201876000000
H	-10.153309000000	-0.767636000000	0.054557000000
C	-12.238006000000	-2.514790000000	0.143485000000
C	-13.314957000000	-3.397525000000	0.155511000000
S	-12.809222000000	-0.826787000000	0.157128000000
C	-14.561561000000	-2.746839000000	0.173537000000
H	-13.168341000000	-4.468962000000	0.151514000000

C	-14.453530000000	-1.360084000000	0.176746000000
C	-16.028157000000	-3.176958000000	0.192114000000
C	-15.762815000000	-0.783343000000	0.196474000000
C	-16.706459000000	-1.807206000000	0.205905000000
C	-16.364760000000	-3.989471000000	1.462487000000
C	-16.399626000000	-3.982446000000	-1.072968000000
S	-16.482942000000	0.787232000000	0.222319000000
C	-18.024972000000	-1.329535000000	0.231330000000
H	-17.433635000000	-4.223103000000	1.494105000000
H	-15.811039000000	-4.933542000000	1.469021000000
H	-16.106684000000	-3.432169000000	2.367108000000
H	-15.845783000000	-4.926062000000	-1.100236000000
H	-17.468882000000	-4.216546000000	-1.076052000000
H	-16.167230000000	-3.419811000000	-1.981239000000
C	-18.107708000000	0.063853000000	0.241092000000
H	-18.899752000000	-1.968078000000	0.247052000000
C	-19.312735000000	0.866760000000	0.259635000000
C	-20.587534000000	0.293884000000	0.216036000000
C	-19.327017000000	2.301716000000	0.325674000000
H	-20.682929000000	-0.785901000000	0.155909000000
N	-21.763046000000	0.950756000000	0.239561000000
C	-20.583798000000	3.022374000000	0.349878000000
N	-18.259716000000	3.104670000000	0.371043000000
C	-21.822239000000	2.282016000000	0.306427000000
N	-20.437403000000	4.348290000000	0.413229000000
S	-18.822370000000	4.648540000000	0.438959000000
C	-23.125565000000	2.893490000000	0.330566000000
C	-23.494116000000	4.235705000000	0.401265000000
S	-24.540838000000	1.826360000000	0.265291000000
C	-24.886930000000	4.409902000000	0.403404000000
H	-22.759172000000	5.028318000000	0.447884000000
C	-25.584724000000	3.203191000000	0.333959000000
C	-25.847814000000	5.595691000000	0.464511000000
C	-26.987893000000	3.475421000000	0.341151000000
C	-27.182906000000	4.855305000000	0.415399000000
C	-25.673547000000	6.534387000000	-0.750575000000
C	-25.686596000000	6.389367000000	1.780718000000
S	-28.471920000000	2.589859000000	0.282220000000
C	-28.539603000000	5.215204000000	0.425482000000
H	-24.682798000000	6.999048000000	-0.734034000000
H	-25.782796000000	5.987851000000	-1.691245000000
H	-26.422893000000	7.331617000000	-0.725489000000
H	-24.694515000000	6.848989000000	1.829227000000
H	-26.433481000000	7.187315000000	1.838258000000
H	-25.808629000000	5.739223000000	2.651300000000
C	-29.384551000000	4.108984000000	0.358724000000
H	-28.923738000000	6.225159000000	0.477801000000
C	-30.822537000000	4.028811000000	0.342106000000
C	-31.694531000000	5.179368000000	0.396711000000
N	-31.374321000000	2.817428000000	0.270787000000
C	-33.132757000000	4.989220000000	0.371540000000
N	-31.324942000000	6.459977000000	0.470990000000
C	-32.710981000000	2.653038000000	0.248577000000
C	-33.679198000000	3.658262000000	0.293178000000
N	-33.812222000000	6.139736000000	0.427895000000
S	-32.703673000000	7.351536000000	0.506159000000
H	-33.029069000000	1.614685000000	0.189029000000
C	-35.098489000000	3.380445000000	0.263262000000
C	-36.167209000000	4.272422000000	0.298962000000
S	-35.685137000000	1.700427000000	0.167552000000
C	-37.419841000000	3.634753000000	0.250822000000
H	-36.010632000000	5.340835000000	0.357523000000
C	-37.324441000000	2.249085000000	0.178280000000
C	-38.882512000000	4.078508000000	0.257481000000
C	-38.639026000000	1.685968000000	0.133448000000
C	-39.573416000000	2.717385000000	0.177460000000
C	-39.215025000000	4.956252000000	-0.969967000000
C	-39.242920000000	4.824264000000	1.561802000000
S	-39.372850000000	0.124710000000	0.038359000000
C	-40.896164000000	2.252775000000	0.135053000000

H	-40.282380000000	5.198170000000	-0.988006000000
H	-38.655258000000	5.895924000000	-0.929857000000
H	-38.961895000000	4.443743000000	-1.902059000000
H	-38.677067000000	5.758236000000	1.634791000000
H	-40.309098000000	5.071482000000	1.578004000000
H	-39.016756000000	4.214266000000	2.440589000000
C	-40.991187000000	0.862246000000	0.058162000000
H	-41.765318000000	2.898745000000	0.157635000000
C	-42.203139000000	0.071602000000	-0.001001000000
C	-43.473270000000	0.655303000000	0.023502000000
C	-42.228921000000	-1.361974000000	-0.091465000000
H	-43.560946000000	1.735153000000	0.092366000000
N	-44.653942000000	0.008705000000	-0.031245000000
C	-43.491078000000	-2.070798000000	-0.150326000000
N	-41.167966000000	-2.173635000000	-0.131200000000
C	-44.723320000000	-1.320266000000	-0.117705000000
N	-43.354846000000	-3.396923000000	-0.233425000000
S	-41.742582000000	-3.710702000000	-0.235537000000
C	-46.032094000000	-1.920517000000	-0.175418000000
C	-46.409369000000	-3.258175000000	-0.267370000000
S	-47.439191000000	-0.842411000000	-0.129730000000
C	-47.804207000000	-3.420538000000	-0.301290000000
H	-45.680324000000	-4.056602000000	-0.306228000000
C	-48.492335000000	-2.209466000000	-0.235396000000
C	-48.772764000000	-4.597970000000	-0.394786000000
C	-49.898877000000	-2.470453000000	-0.277137000000
C	-50.102437000000	-3.846958000000	-0.369070000000
C	-48.633215000000	-5.548332000000	0.815790000000
C	-48.587452000000	-5.381475000000	-1.713879000000
S	-51.375414000000	-1.572911000000	-0.244186000000
C	-51.463168000000	-4.194161000000	-0.413992000000
H	-47.646085000000	-6.020895000000	0.817657000000
H	-48.759421000000	-5.008826000000	1.758385000000
H	-49.388019000000	-6.339253000000	0.766855000000
H	-47.597537000000	-5.847291000000	-1.744369000000
H	-49.337943000000	-6.173973000000	-1.794806000000
H	-48.685818000000	-4.723149000000	-2.581302000000
C	-52.297452000000	-3.081996000000	-0.356442000000
H	-51.855106000000	-5.200114000000	-0.484988000000
C	-53.739868000000	-2.988829000000	-0.373848000000
C	-54.609579000000	-4.144797000000	-0.460948000000
N	-54.276445000000	-1.775210000000	-0.305018000000
C	-56.042732000000	-3.939125000000	-0.469712000000
N	-54.251120000000	-5.428997000000	-0.537242000000
C	-55.629146000000	-1.606890000000	-0.315124000000
C	-56.555803000000	-2.617930000000	-0.393443000000
N	-56.744690000000	-5.078276000000	-0.553071000000
S	-55.644313000000	-6.299421000000	-0.614183000000
H	-55.959422000000	-0.573279000000	-0.254569000000
H	-57.621495000000	-2.425089000000	-0.397402000000

Oligomer 2 in vacuum

Atom	X	Y	Z
C	-0.569466642024	-59.398983875201	-0.102798912355
C	-1.245397605741	-58.310174014385	0.368368338328
C	-0.425352751795	-57.152701278859	0.393318059574
C	0.848925873039	-57.397629764816	-0.059808123151
S	1.075200084471	-59.037692591476	-0.524573735526
H	-0.940057528754	-60.405860054531	-0.230524859278
H	-2.282191223760	-58.348460304579	0.679859466695
C	1.612636967649	-56.174755278596	-0.001691856878
C	0.803036775245	-55.172285138290	0.490042477452
S	3.200208581428	-55.641227520972	-0.364345824231
C	1.468422377480	-53.935629202836	0.581915649115
C	2.778840129240	-54.018647067240	0.156597301245
H	1.038063384323	-53.009506943544	0.937978220534
C	-0.593125258976	-55.694953614136	0.796090942772
C	-0.931950158643	-55.558614502672	2.288552377041
H	-0.974474469996	-54.504521954658	2.577263932606

H	-1.906423112501	-56.006318352928	2.504362862911
H	-0.181283325466	-56.054237485702	2.907940859004
C	-1.658490010124	-54.993252996137	-0.060323620439
H	-2.644838780250	-55.431723230485	0.117073560693
H	-1.712735839701	-53.930047999427	0.190525485542
H	-1.428452743265	-55.083730134430	-1.124128868901
C	3.801633296645	-52.999088013779	0.085693338429
C	3.589744262746	-51.628769444333	0.481149697679
C	4.675737400401	-50.690745388499	0.371223703419
C	5.950976715147	-51.115321310409	-0.125339857468
C	7.100941218957	-50.231213528863	-0.267862521882
C	8.353228849082	-50.577834937008	-0.736938588095
S	7.037085884510	-48.526749877809	0.160148815172
C	9.250646449223	-49.494984321638	-0.754296244401
H	8.606303754981	-51.581200975580	-1.055122004030
C	8.677814804729	-48.328602250254	-0.298326788518
C	9.659226711914	-47.276378879106	-0.348743649603
C	10.841950058000	-47.795080825804	-0.837581936103
S	9.759810201928	-45.605290746464	0.020597972157
C	11.862042126882	-46.830804663966	-0.921006136800
C	11.438153444426	-45.588296572357	-0.492648756535
H	12.869997601685	-47.003383745951	-1.273040536191
C	10.703840736684	-49.278062018885	-1.150274204142
C	12.156139347812	-44.336122093299	-0.413898462459
C	13.536060340507	-44.182411092535	-0.802448002567
C	12.109532892090	-42.072920984287	0.142899390622
C	14.158342552604	-42.890023918723	-0.684471869192
C	13.414041677273	-41.771358166399	-0.186863689378
H	11.460041802692	-41.294867489202	0.529512951877
N	2.473061245707	-51.088715987770	0.956460095287
N	4.345118838033	-49.466324104666	0.768259227544
N	14.350510116341	-45.117969610164	-1.277790609392
N	15.428424781967	-42.888759666713	-1.075703581510
N	11.510785328921	-43.282628188549	0.037521505435
S	2.788178207558	-49.518412058098	1.237103706260
S	15.785849840515	-44.403583137700	-1.549027380430
C	10.930510395133	-49.563703934835	-2.642790938283
H	10.753809719014	-50.620411855180	-2.863526596126
H	10.258961484126	-48.966158273809	-3.263061992809
H	11.960440309539	-49.328535608731	-2.925842884500
C	11.654962618136	-50.126725844490	-0.292537183623
H	11.490059026369	-51.192605697911	-0.474983427795
H	12.696618262821	-49.900675834559	-0.537554035915
H	11.502552412559	-49.932631652274	0.771358264485
C	6.000267492686	-52.451209239049	-0.462452332329
H	6.921489994808	-52.873547857474	-0.849315284922
N	4.987741406027	-53.345195502195	-0.364533471140
C	13.967326252523	-40.431618152413	-0.036171118632
C	13.304654959735	-39.315263875180	0.436353594331
S	15.630721123893	-40.046556930734	-0.457421010915
C	14.115733540903	-38.166625888262	0.461507411760
H	12.269166205645	-39.334186099196	0.751643061571
C	15.392583015055	-38.413133179653	0.008055494022
C	13.944847638971	-36.709167460123	0.864068034751
C	16.152176511618	-37.191460681892	0.066709427752
C	15.341313123224	-36.187505383169	0.558051861931
C	13.605603295782	-36.572008722314	2.356289789988
C	12.880032932564	-36.008068509742	0.006597992604
S	17.740201434119	-36.656270205159	-0.295218893689
C	16.005567746939	-34.951518257427	0.649587402142
H	13.563444764948	-35.517754864745	2.644294093597
H	12.630812716317	-37.019017487920	2.572040007929
H	14.355689494721	-37.067608508427	2.976392112051
H	11.893443047050	-36.445902853742	0.184145098742
H	12.826117103206	-34.944671481787	0.256507779204
H	13.110170624163	-36.099551669023	-1.057095010710
C	17.316931234805	-35.034251562226	0.224809399925
H	15.574730556057	-34.025363639605	1.004922439311
C	18.338327279356	-34.013716477789	0.154391547931
C	18.124847909872	-32.643559494755	0.549221081724

N	19.525268564641	-34.359088656143	-0.295014980680
C	19.210132530091	-31.704653451542	0.439514872864
N	17.007467468449	-32.104234580200	1.023707137280
C	20.536844680957	-33.464533875567	-0.392618337595
C	20.486019644601	-32.128261263155	-0.056130079550
N	18.878241329166	-30.480353308898	0.835902632398
S	17.321127668527	-30.533514819342	1.303923434950
H	21.458806515980	-33.886139853105	-0.778509805990
C	21.635049039413	-31.243277429283	-0.198448328521
C	22.887589252146	-31.588664231642	-0.668068161411
S	21.570057999580	-29.539101709880	0.230691139387
C	23.784170994017	-30.505236900887	-0.684792759971
H	23.141285485779	-32.591590295091	-0.987138041078
C	23.210551313817	-29.339536954935	-0.227861434477
C	25.237111657259	-30.286927424705	-1.080946877558
C	24.191125216093	-28.286569115595	-0.277807617726
C	25.374135110391	-28.804057532415	-0.767265155175
C	25.463662961804	-30.571361121037	-2.573712275580
C	26.189076130875	-31.135442561628	-0.223997875169
S	24.290527042549	-26.615640906902	0.092632001948
C	26.393477413019	-27.838951482991	-0.850275220058
H	25.287742686033	-31.628055385474	-2.795132031004
H	24.791510758025	-29.973916445870	-3.193425822834
H	26.493345334524	-30.335192288312	-2.856826463704
H	26.024961096445	-32.201321770023	-0.407151232087
H	27.230498990680	-30.908409653779	-0.469088546076
H	26.036751323097	-30.942206726403	0.840065424124
C	25.968714755904	-26.597064033273	-0.420994502241
H	27.401485269891	-28.010508948512	-1.202656165150
C	26.685774850389	-25.344367477487	-0.341627416402
C	28.065476562848	-25.189371059628	-0.730376403511
N	26.039703966753	-24.291667477192	0.110589094767
C	28.686740850977	-23.896548437354	-0.611869091487
N	28.880582181583	-26.124040227879	-1.206360693980
C	26.637504689372	-23.081542483533	0.216546140581
C	27.941655259119	-22.778728359746	-0.113519646490
N	29.956746267625	-23.894073994386	-1.003351532221
S	30.315292798296	-25.408359590855	-1.477504744504
H	25.987546938625	-22.304279778784	0.603959461495
C	28.493867361729	-21.438600957414	0.037565524503
C	27.830010125203	-20.322663962253	0.509430478281
S	30.157419445257	-21.052460307788	-0.382080174126
C	28.640342410126	-19.173522439198	0.535452181663
H	26.794177054062	-20.342213164469	0.823549985957
C	29.917802138280	-19.419231804679	0.083275877177
C	28.468143017413	-17.716147979485	0.937774166736
C	30.676554054323	-18.197074562855	0.142587869816
C	29.864576600748	-17.193613229812	0.633083605501
C	28.127367653119	-17.579116510742	2.429660297518
C	27.403719738079	-17.015759932071	0.079235638667
S	32.264589671716	-17.660911222072	-0.217835717019
C	30.527961627050	-15.957188049169	0.725170087154
H	28.084289460389	-16.524872136208	2.717560330369
H	27.152640294878	-18.026703889268	2.644491624079
H	28.877153912900	-18.074221029876	3.050520611762
H	26.417227829439	-17.454187258733	0.255860630102
H	27.348909452719	-15.952381991118	0.329030161345
H	27.634935484226	-17.107161637471	-0.984230625701
C	31.839775902210	-16.039125077546	0.301645864919
H	30.096194856599	-15.031275992013	1.080009957615
C	32.860613603978	-15.017963438801	0.232043228887
C	32.645963544501	-13.647878662808	0.626462739609
N	34.048170540134	-15.362709782617	-0.216205878618
C	33.730749444815	-12.708311692220	0.517446298542
N	31.527866562103	-13.109168890240	1.099961920956
C	35.059295574933	-14.467571039725	-0.313069870974
C	35.007303202657	-13.131218346450	0.022922892383
N	33.397778724456	-11.484148079324	0.913353717934
S	31.840331309570	-11.538207969199	1.380159426602
H	35.981921760726	-14.888749158772	-0.697834597443

C	36.155858456871	-12.245534144360	-0.118833825160
C	37.408493938622	-12.589946776224	-0.588908470692
S	36.090184954241	-10.541782817937	0.311884982590
C	38.304639110283	-11.506152575902	-0.604616406458
H	37.662533905027	-13.592419847603	-0.909125407087
C	37.730594837321	-10.341118951957	-0.146515028980
C	39.757426474800	-11.286883914510	-1.000774271905
C	38.710725379877	-9.287686250241	-0.195665545089
C	39.893873721837	-9.804252206050	-0.685757123801
C	39.983872652302	-11.569856912931	-2.493832370526
C	40.709864031928	-12.135790568052	-0.144740261982
S	38.809528937453	-7.617039768535	0.176252079981
C	40.912819385095	-8.838670135706	-0.768056087601
H	39.808341729642	-12.626418574737	-2.716194226658
H	39.311388494729	-10.972113510282	-3.112897312644
H	41.013418309560	-11.333017035407	-2.776883299440
H	40.546159448350	-13.201568978686	-0.328845379749
H	41.751157915888	-11.908104774862	-0.389773208351
H	40.557612793362	-11.943589844686	0.919520964617
C	40.487625313991	-7.597333259769	-0.337629052966
H	41.920837394781	-9.009511640859	-1.120751626043
C	41.204244568608	-6.344431827894	-0.257354523911
C	42.583830759676	-6.188673760647	-0.646206114668
N	40.557892421050	-5.292304847866	0.195779976415
C	43.204674090362	-4.895735204770	-0.526809357288
N	43.399176830702	-7.122699525551	-1.123047081358
C	41.155299517865	-4.082048868938	0.302563011505
C	42.459296032752	-3.778541509712	-0.027482947156
N	44.474612434790	-4.892529969621	-0.918504800595
S	44.833599315386	-6.406323008181	-1.393874971099
H	40.505135221167	-3.305295338988	0.690651450039
C	43.011121607460	-2.438359400636	0.124502531445
C	42.347095683289	-1.322994015799	0.597490274364
S	44.674402721606	-2.051368481046	-0.295450483626
C	43.157071492084	-0.173608185145	0.624076187721
H	41.311390162560	-1.343120046384	0.911998477975
C	44.434428417652	-0.418578445637	0.171211943281
C	42.984606310654	1.283423816370	1.027553731338
C	45.192839926870	0.803756025578	0.231134476484
C	44.380769676636	1.806605618966	0.722698073255
C	42.644381084740	1.419257293780	2.519675019897
C	41.919640212133	1.984138363287	0.169955382801
S	46.780576594057	1.340637911188	-0.129532726089
C	45.043845452418	3.043167227174	0.815435046839
H	42.601127034463	2.473277100610	2.808370531335
H	41.669861906747	0.971242816283	2.734564129693
H	43.394551324125	0.923903777121	3.139872657140
H	40.933343492211	1.545298457351	0.346648222654
H	41.864627169173	3.047316129477	0.420554945557
H	42.150460642209	1.893589026691	-0.893669745115
C	46.355499174435	2.961914188738	0.391307260047
H	44.611965117289	3.968698475347	1.171131486992
C	47.376066104852	3.983391342639	0.322010036241
C	47.161286709068	5.353133508149	0.717505097034
N	48.563486272890	3.639236093706	-0.127033934976
C	48.245857134259	6.292990871163	0.608794896044
N	46.043245092814	5.891279208221	1.191784521560
C	49.574384440910	4.534653038055	-0.223692787380
C	49.522315777235	5.870707579862	0.113481200715
N	47.912780002912	7.516803381527	1.005701626328
S	46.355509650598	7.462099953136	1.473004773539
H	50.496858690320	4.114002631319	-0.609392724074
C	50.670697588851	6.756662688229	-0.027915115887
C	51.923657702408	6.412437725762	-0.497262346212
S	50.604333417725	8.460576263717	0.402076374991
C	52.819441861342	7.496520688252	-0.513149766671
H	52.178209014308	5.409865353205	-0.816764486318
C	52.244832395965	8.661590435504	-0.055848410328
C	54.272340328033	7.716064065195	-0.908741615712
C	53.224680103264	9.715306218538	-0.105008168603

C	54.408181518596	9.198878275801	-0.594339479277
C	54.499561108904	7.432482836402	-2.401565355213
C	55.224652387911	6.867849970137	-0.051883781137
S	53.322853859095	11.386163032612	0.266207721422
C	55.426900115415	10.164733496904	-0.676640670675
H	54.324473422319	6.375762725235	-2.623529558676
H	53.827166671216	8.029730713476	-3.021205870473
H	55.529158136737	7.669532713588	-2.684254032072
H	55.061375688055	5.801933206991	-0.235577194540
H	56.265986950819	7.095760872534	-0.296535501867
H	55.071845740264	7.060491006890	1.012218254509
C	55.001156655349	11.406117883961	-0.246966765327
H	56.435122798403	9.994035451980	-1.028818399684
C	55.717410990234	12.659291808083	-0.166934348275
C	57.097087509309	12.815264934844	-0.555309621800
N	55.070592146537	13.711408117071	0.285498246523
C	57.717522267605	14.108430603547	-0.436188919645
N	57.912880156797	11.881268939032	-1.031466040323
C	55.667601772859	14.921895749829	0.392034108275
C	56.971654142091	15.225640436508	0.062399556343
N	58.987612274826	14.111827760694	-0.827400421087
S	59.347204616585	12.597960153204	-1.302018597653
H	55.017055098090	15.698620477201	0.779529069897
C	57.523062378255	16.566013049580	0.214080187972
C	56.858570396004	17.681346473765	0.686482421448
S	59.186363090868	16.953417009371	-0.205492544080
C	57.668167792526	18.830998469244	0.712911129313
H	55.822778974532	17.661018977019	1.000704824498
C	58.945736027903	18.586284866153	0.260516474826
C	57.495164797754	20.288118237674	1.115837144625
C	59.703781211754	19.808906386698	0.320251691952
C	58.891258205679	20.811602683070	0.811212436906
C	57.154472574746	20.424380563532	2.607812027125
C	56.430253265120	20.988248876663	0.257695674161
S	61.291476366677	20.346263884365	-0.040092330280
C	59.553904913032	22.048488664970	0.903755680645
H	57.110884523183	21.478489456188	2.896129505437
H	56.180001304923	19.976199188046	2.822586095327
H	57.904597482485	19.929434307046	3.228390118162
H	55.444012003841	20.549228705511	0.434270451257
H	56.374911084419	22.051504552432	0.507890078229
H	56.661401085329	20.897377145637	-0.805831207014
C	60.865648915973	21.967530232728	0.480081482605
H	59.121614872314	22.973995846340	1.259009428150
C	61.885950301751	22.989472523275	0.410783031380
C	61.670422267731	24.359195799216	0.805791165416
N	63.073574854123	22.645673013590	-0.037719559439
C	62.754613581773	25.299475284089	0.697109246906
N	60.551975845855	24.896989666236	1.279581775154
C	64.084217319505	23.541562541031	-0.134324209152
C	64.031428917353	24.877638325185	0.202325340718
N	62.420840734211	26.523269556448	1.093559729234
S	60.863527454798	26.468025543967	1.560399911787
H	65.007023375146	23.121161047411	-0.519480436139
C	65.179534232930	25.764224496668	0.060964217879
C	66.432472810168	25.420669539620	-0.408498234413
S	65.112517423418	27.467968426343	0.491351581050
C	67.327922341255	26.505358967856	-0.424079655377
H	66.687468056524	24.418315237269	-0.728364206740
C	66.752799856841	27.669708507329	0.033498110570
C	68.780742602529	26.725744831050	-0.819669911053
C	67.732571852755	28.724436872987	-0.015406457003
C	68.915719845916	28.208532326551	-0.504778913751
C	69.008011996715	26.442599387870	-2.312568288922
C	69.733448385183	25.877629564051	0.036846658736
S	67.829706118098	30.394994416345	0.356312003596
C	69.934123979240	29.175823746247	-0.586625086226
H	68.833465360977	25.385838743502	-2.534858297203
H	68.335274921901	27.039703208505	-2.931981065610
H	70.037468318287	26.680283713975	-2.595257576911

H	69.570724551659	24.811667326418	-0.147184531297
H	70.774673532309	26.106156423294	-0.207720465937
H	69.580548796636	26.069873049434	1.101010394401
C	69.507285812798	30.415596793628	-0.156716737644
H	70.942603550619	29.006384873859	-0.938757301690
C	70.223286882083	31.672819944821	-0.075499614857
C	71.608178812693	31.817909238219	-0.468204860182
N	69.570293896707	32.717734316081	0.377342681056
C	72.229797318937	33.109219926860	-0.349669801927
N	72.420889342301	30.881088060495	-0.944365901826
C	70.177930885503	33.935636605489	0.482547885453
C	71.470716439131	34.201658375502	0.147350017107
N	73.502332172456	33.118654564329	-0.740302474607
S	73.855788745542	31.601856613231	-1.214110787040
H	69.539962992187	34.724650394421	0.869224905687
H	71.908202237035	35.186178765046	0.248977386033

Oligomer 2 in solvent

Atom	X	Y	Z
C	-58.946250551995	-0.708406842536	-1.399067769438
C	-58.480436615358	-1.943175967567	-1.749648809172
C	-57.064540309819	-1.961298775764	-1.853018595394
C	-56.497822805818	-0.739519035547	-1.577830541232
S	-57.673457252709	0.455494281835	-1.187954764226
H	-59.971690114610	-0.402372084348	-1.248690949726
H	-59.130259191705	-2.792324706454	-1.922930402666
C	-55.064863310791	-0.848567398366	-1.704914026544
C	-54.749133139240	-2.143051378484	-2.060012262770
S	-53.685128590356	0.157282988097	-1.539922529828
C	-53.362917330626	-2.343169529144	-2.206671379165
C	-52.641490153868	-1.193292937587	-1.959266006563
H	-52.886419268992	-3.275003468083	-2.479017262478
C	-56.002254190653	-2.996273401040	-2.193063553986
C	-56.173122181080	-3.529826325842	-3.624095615546
H	-55.358517959548	-4.214432387589	-3.876044083449
H	-57.115496083811	-4.076631897885	-3.717916140278
H	-56.174363380267	-2.713777177204	-4.350347541042
C	-56.004735430086	-4.157571337247	-1.186713469336
H	-56.944956533914	-4.712772173675	-1.246563496095
H	-55.187856294513	-4.851735756500	-1.402609283519
H	-55.884882550247	-3.792080553640	-0.164244011096
C	-51.214232842391	-0.959110884898	-1.995557758277
C	-50.249347776636	-1.979762728193	-2.320275314197
C	-48.852537515889	-1.643061391790	-2.345158122042
C	-48.430110018458	-0.308532503750	-2.043836165007
C	-47.040689037176	0.131192565081	-2.068476448716
C	-46.594615181785	1.435216473262	-2.129254830697
S	-45.696627283443	-0.996794481600	-1.959658666701
C	-45.190345470420	1.533949440562	-2.068103469996
H	-47.264374411440	2.280099140482	-2.230308570115
C	-44.577344188915	0.305160134964	-1.968434382584
C	-43.150663403784	0.497450280408	-1.936398475359
C	-42.883032497517	1.849347220358	-2.009773823626
S	-41.732464842896	-0.461920444081	-1.824377399255
C	-41.504736558498	2.132375456055	-1.974455653451
C	-40.740359384342	0.987445775395	-1.875330409730
H	-41.062890509270	3.118432196524	-2.017878797576
C	-44.167411150140	2.659481193318	-2.106645290988
C	-39.303856750701	0.826242274135	-1.812748019707
C	-38.378815368053	1.931453186439	-1.831779034438
C	-37.489959544111	-0.632099723247	-1.666464619149
C	-36.967459580664	1.665994224969	-1.781328366809
C	-36.492617704577	0.317174575760	-1.708514378981
H	-37.224902228298	-1.679629788158	-1.567317675326
N	-50.489025379404	-3.253329056533	-2.615463355744
N	-48.075243136101	-2.675704187921	-2.659376502976
N	-38.668728111017	3.227174345374	-1.893025868003
N	-36.229914517936	2.772680765500	-1.805779068560
N	-38.824108920338	-0.396478067397	-1.728353302423

S	-49.051724183638	-3.955087987817	-2.897452886426
S	-37.257685631415	4.031335710409	-1.884407014332
C	-44.328155252288	3.610752772788	-0.910486619714
H	-45.290726718111	4.127297840349	-0.959839622963
H	-44.277515179775	3.065994211559	0.035038366770
H	-43.538155342283	4.366733980174	-0.915723758201
C	-44.242614253841	3.443430868741	-3.426291015037
H	-45.204344465524	3.956817016644	-3.511342923093
H	-43.452016574840	4.197681896806	-3.467112878871
H	-44.129496705845	2.778815815443	-4.285837584889
C	-49.457742913052	0.550570996795	-1.722843061733
H	-49.229155886873	1.572557290788	-1.438816575656
N	-50.780373473246	0.250515415563	-1.711281719778
C	-35.082135509050	-0.048536576628	-1.683476539732
C	-34.552151585274	-1.290174675288	-1.967354891993
S	-33.826187457219	1.087108257541	-1.211352993762
C	-33.155186834876	-1.339797116058	-1.789220753576
H	-35.156955231652	-2.121625298148	-2.307243885895
C	-32.631541543782	-0.134939241171	-1.376829257387
C	-32.063598955842	-2.385725036522	-1.961700181996
C	-31.203597412927	-0.267287932609	-1.247649885676
C	-30.845711086228	-1.559190560788	-1.575492490426
C	-31.975966454649	-2.873862965489	-3.416315950929
C	-32.264835493646	-3.576290153663	-1.010932233090
S	-29.863167729662	0.708122233490	-0.804881038716
C	-29.459638157068	-1.781244572570	-1.471924164843
H	-31.137982496114	-3.566129399953	-3.535779890020
H	-32.891979066872	-3.400192544502	-3.698613629194
H	-31.832897455617	-2.037596514525	-4.104440431881
H	-33.184997576304	-4.112083693169	-1.259741935094
H	-31.431032188249	-4.278350522722	-1.097542567985
H	-32.329335369891	-3.244417865711	0.027836143405
C	-28.780193441958	-0.650747097120	-1.065430604500
H	-28.953848865116	-2.714078762754	-1.680389460070
C	-27.366392592547	-0.440101677938	-0.841410033781
C	-26.371581021957	-1.468112308336	-1.015360532554
N	-26.975605905964	0.756291876420	-0.456141036768
C	-24.988193731472	-1.151465472600	-0.789090577012
N	-26.570105476600	-2.731976334645	-1.375894451215
C	-25.668523699708	1.037257926755	-0.230582415466
C	-24.609360732990	0.170266929723	-0.389186155670
N	-24.179728934780	-2.189249498930	-0.985799261464
S	-25.113664699742	-3.449165799034	-1.418306442241
H	-25.483049804145	2.049952172564	0.111775904236
C	-23.231937554622	0.590251028031	-0.165107232923
C	-22.774459527396	1.889504683318	-0.083652711420
S	-21.926691588720	-0.560214979391	0.089582001496
C	-21.396853675743	1.965692457369	0.200021318466
H	-23.414821837545	2.748443260098	-0.240271906004
C	-20.812756617150	0.725055998659	0.325121301683
C	-20.375841029494	3.078228505270	0.384438340431
C	-19.407193196941	0.896652943874	0.587261036125
C	-19.125431002842	2.247062761439	0.630485192613
C	-20.714980211641	3.961022538591	1.595772844149
C	-20.243505685836	3.937874504551	-0.882453379299
S	-18.026220741324	-0.084864008867	0.860763613808
C	-17.767588648562	2.510554440209	0.889787419663
H	-21.656370931386	4.491903603937	1.429684004398
H	-20.812605515718	3.362155729006	2.504161578609
H	-19.930499311864	4.705651200827	1.756222183170
H	-21.178390171107	4.468472156610	-1.083120601374
H	-19.452480234601	4.682203834417	-0.756792652317
H	-20.002643260866	3.322658314035	-1.752403085209
C	-17.031658433277	1.352583995799	1.039467449450
H	-17.320331715862	3.492067415348	0.966990676556
C	-15.621575606274	1.172099584686	1.308997177377
C	-14.696883615008	2.266778693880	1.464416686512
N	-15.165265954173	-0.058263975939	1.410558299892
C	-13.316047677645	1.984434313092	1.744261358203
N	-14.961584836331	3.566593304644	1.380131923629

C	-13.856295694040	-0.310843245949	1.660029227317
C	-12.869341617468	0.629021282492	1.861117354977
N	-12.575637895111	3.083030366474	1.863611690476
S	-13.564496644225	4.354340776135	1.635135757531
H	-13.602071464958	-1.365343980974	1.687922774758
C	-11.496014623174	0.249335129800	2.167268709111
C	-11.066560253687	-0.978964208718	2.625901253314
S	-10.143933226010	1.346394498782	1.924480896045
C	-9.665847968072	-1.049878424671	2.763081245717
H	-11.747653163294	-1.785507375772	2.867117521829
C	-9.040357141166	0.125977832264	2.414015291946
C	-8.659533469604	-2.093589771742	3.225462950531
C	-7.621984224982	-0.024811685295	2.608682950085
C	-7.370117444493	-1.299444249210	3.073861985173
C	-8.670780405887	-3.332362506361	2.316138995013
C	-8.903572433973	-2.502028914788	4.686720705071
S	-6.198364199027	0.913317943158	2.416834506701
C	-5.999017084271	-1.538557470058	3.285055107208
H	-7.890638073041	-4.035512437690	2.620461476623
H	-9.633751585112	-3.846149688127	2.382592477974
H	-8.498547516511	-3.056803653157	1.273181601251
H	-9.870524170991	-3.002835874975	4.786743899769
H	-8.127518763640	-3.194871484616	5.023420304947
H	-8.896925004663	-1.630710954475	5.345585530348
C	-5.225116891302	-0.438117758838	2.976647731343
H	-5.568962236406	-2.462824731209	3.646025816452
C	-3.792079678025	-0.252188009521	3.049824808603
C	-2.877242443171	-1.278373943292	3.482032182856
N	-3.304994944713	0.918279463552	2.696063119670
C	-1.470809136310	-0.987686492135	3.535237545472
N	-3.172915345710	-2.518698809257	3.857258104989
C	-1.974137217047	1.174238975084	2.736136496379
C	-0.989783766379	0.306527954679	3.156059058580
N	-0.742798296134	-2.020754122919	3.950049280032
S	-1.772566042432	-3.246246587870	4.241130093305
H	-1.699773337191	2.164896101608	2.389013027741
C	0.412815897122	0.699576394171	3.199686386012
C	0.903690282458	1.988440669331	3.162594034520
S	1.718388969045	-0.477111894522	3.248688680827
C	2.311535151372	2.035224065856	3.151296725721
H	0.262460287306	2.860942127890	3.158800098432
C	2.883345165149	0.783185795125	3.187630622434
C	3.371376834323	3.126168379810	3.126288661568
C	4.316421863535	0.926037441279	3.193269376094
C	4.628597674874	2.269842404984	3.153531884323
C	3.277293511932	4.031793639217	4.364341086238
C	3.286637081173	3.966228345889	1.842329105650
S	5.704097266561	-0.083450355383	3.222547547803
C	6.016090451351	2.505035789794	3.144597266912
H	2.331681985910	4.581042387005	4.364980895343
H	3.336693990402	3.446990608694	5.285109036171
H	4.091829063600	4.761248275297	4.365871731780
H	2.341244369658	4.514537138399	1.806638756634
H	4.101419826745	4.694704395753	1.808619809015
H	3.352343836638	3.334397435254	0.953620586496
C	6.743425342228	1.332587448924	3.179054917964
H	6.489884881273	3.476608594303	3.114860284489
C	8.175160698290	1.123432304168	3.184583044432
C	9.133958737675	2.198281703561	3.130679040297
N	8.618096823833	-0.115014109971	3.234244928807
C	10.536862860301	1.888752663936	3.153657883013
N	8.883837881572	3.501710885934	3.057518228324
C	9.945444891380	-0.393708793346	3.240779725445
C	10.970906467338	0.526113106294	3.225062208462
N	11.308128681897	2.970991004766	3.096880242551
S	10.319280815284	4.260382313499	3.019554252236
H	10.179526404885	-1.453162664921	3.251240942577
C	12.369852014103	0.121061000312	3.277521058128
C	12.855357155854	-1.106228477206	3.679769884970
S	13.671862316756	1.180485519466	2.754867439888

C	14.255185732732	-1.206629282319	3.552284622438
H	12.217113108942	-1.891184485194	4.066057196567
C	14.824999240713	-0.053940476735	3.059862794130
C	15.311247839481	-2.263476224539	3.841626997928
C	16.251420025400	-0.233467340703	2.985877183850
C	16.563143326106	-1.502883387317	3.428554484195
C	15.103899026439	-3.519390671013	2.980718781100
C	15.342332240330	-2.636111924154	5.332159840048
S	17.629972130195	0.666696009020	2.503435234944
C	17.944876600131	-1.768594667428	3.384105552125
H	15.915375605140	-4.233428769454	3.146108112743
H	14.162560462767	-4.010728799999	3.241970560714
H	15.077906136860	-3.267786095248	1.917994849542
H	14.404146989269	-3.114833002493	5.626096244980
H	16.156955872918	-3.337333339277	5.532885067055
H	15.487998103019	-1.751171291847	5.955859697085
C	18.666602457504	-0.693528636207	2.906246290688
H	18.418620976324	-2.693860561282	3.682313007272
C	20.090791323472	-0.538988179310	2.703032159479
C	21.053443847060	-1.570606873340	2.996137981141
N	20.522342586691	0.607273597570	2.220965534410
C	22.449628917132	-1.310754884096	2.775742023329
N	20.812926745057	-2.789865953526	3.467656242334
C	21.841204742739	0.833680509854	2.003008193290
C	22.872448322146	-0.041616116414	2.264969493084
N	23.225418400711	-2.345138935818	3.088111737641
S	22.248469275592	-3.535420042451	3.612850876032
H	22.062159241443	1.805510003670	1.574224573314
C	24.264930988017	0.321794546753	2.035126569212
C	24.760206877879	1.597453002571	1.858652752147
S	25.540091346599	-0.880404270549	1.894948425148
C	26.143594982152	1.613017587139	1.593233582782
H	24.142060174410	2.483178444059	1.935807584364
C	26.693906194787	0.350881647389	1.576578447737
C	27.198969632717	2.679250959271	1.340777946947
C	28.107604133225	0.462744873400	1.325774808682
C	28.428313720583	1.797253165920	1.180621670806
C	27.336755581778	3.629381914320	2.540665858751
C	26.904095595286	3.475490263329	0.059902725959
S	29.463533623811	-0.574773151991	1.151900370161
C	29.796315762299	2.002025622596	0.921229266892
H	26.414045568446	4.198250412297	2.684785945025
H	27.547494628369	3.076048196578	3.458672228229
H	28.149792579686	4.340943230208	2.372504077057
H	25.977647729036	4.045990221100	0.169316764949
H	27.713630950230	4.181417361181	-0.144892792126
H	26.800188793481	2.811417397071	-0.801233423022
C	30.500534516378	0.816042121807	0.873703199764
H	30.272390054364	2.961465847630	0.772220656333
C	31.908138817972	0.575946612397	0.640021921840
C	32.860710074786	1.626765804003	0.383403443194
N	32.333033584222	-0.669635903489	0.658575791003
C	34.241795144586	1.287182268137	0.178327490100
N	32.622986860447	2.932334734758	0.307634380949
C	33.637026985261	-0.977339249724	0.448528408581
C	34.659536466030	-0.081698164681	0.224392668211
N	35.009449816343	2.349863164852	-0.047150749673
S	34.042927495708	3.657614085815	-0.000611389173
H	33.852211095127	-2.040761996403	0.458213968312
C	36.040039532762	-0.516764843775	0.054423730210
C	36.564934392859	-1.741641907283	0.412098241837
S	37.254259952618	0.495604244755	-0.714655870905
C	37.921585367367	-1.877787592147	0.056451570215
H	35.987593510378	-2.499905614988	0.926274190810
C	38.421123261995	-0.754420133154	-0.563313350007
C	38.992922616444	-2.948078433014	0.205338385633
C	39.812333740393	-0.968737850579	-0.865854704359
C	40.171169328788	-2.230478161405	-0.437184351876
C	38.623708230901	-4.228616873848	-0.559855533952
C	39.264670418545	-3.269650096631	1.683308276946

S	41.107583392311	-0.115025069126	-1.598967549692
C	41.521393296953	-2.529133623843	-0.701005309316
H	39.438061182654	-4.955867338269	-0.500811048791
H	37.729427751504	-4.686369110368	-0.128143144015
H	38.427487832566	-4.015430897233	-1.613119112010
H	38.378565277260	-3.712451135908	2.146510410629
H	40.086301704636	-3.985548141955	1.772786195936
H	39.530662219659	-2.368210728190	2.240050142511
C	42.172411567340	-1.486006140093	-1.327875848414
H	42.021895849106	-3.455515192941	-0.454268784807
C	43.546273413073	-1.369972675861	-1.767024758910
C	44.521339906719	-2.420438810106	-1.616093951145
N	43.916486294249	-0.243297789973	-2.337780953909
C	45.867472300503	-2.198670887031	-2.067873011755
N	44.334933531274	-3.626337115244	-1.089021410244
C	45.186096539024	-0.053165188978	-2.774437243689
C	46.227445923586	-0.948515409498	-2.666098740711
N	46.661183651943	-3.247294319193	-1.868169696676
S	45.757872796459	-4.405207012798	-1.168187729716
H	45.354334175908	0.904869881773	-3.255145145651
C	47.570711628797	-0.622765008383	-3.128327919586
C	48.062172839509	0.638612402512	-3.394002756858
S	48.771060266536	-1.859368795521	-3.475627087934
C	49.380446997924	0.616122235001	-3.891188597649
H	47.489307636022	1.540431202880	-3.217614357902
C	49.885244825759	-0.660500137626	-3.993917508296
C	50.405417523475	1.651798904409	-4.328976277557
C	51.237708453711	-0.588685649803	-4.484538597924
C	51.564879240071	0.735504566786	-4.691275390423
C	50.774721524235	2.601769071254	-3.178756259694
C	49.914633487510	2.452181342546	-5.545572777458
S	52.514750876322	-1.664734227422	-4.879049009441
C	52.875345275355	0.900349190958	-5.180074806997
H	49.907523169171	3.198061681512	-2.881766367978
H	51.125857505765	2.045762791114	-2.306462609651
H	51.566025179078	3.288512579803	-3.491597425379
H	49.033517510219	3.044163317511	-5.282989263733
H	50.693150527727	3.138641219348	-5.889483400596
H	49.650220116748	1.788873656188	-6.372295241942
C	53.527366687895	-0.304632984411	-5.337160388775
H	53.346433253686	1.845307956858	-5.413652241024
C	54.870548718799	-0.588883203705	-5.804695824177
C	55.798100182944	0.442849601612	-6.209122605818
N	55.244468536623	-1.847360036350	-5.856344901158
C	57.107769312854	0.059673059372	-6.657243007300
N	55.597204126511	1.757007454373	-6.224500181341
C	56.495064419985	-2.192447810351	-6.285896159840
C	57.455579200171	-1.316344295852	-6.691465776640
N	57.868663076073	1.097814167063	-7.002012631966
S	56.971557298284	2.435413665531	-6.767228727059
H	56.694965298207	-3.259469906836	-6.287405562314
H	58.432806443093	-1.641837490942	-7.023398647545

Oligomer 3 in vacuum

Atom	X	Y	Z
C	-10.785492477085	104.241744501127	36.270205588576
C	-10.740165361576	103.041536651048	36.918974812228
C	-11.117411111626	101.968849892563	36.069586439653
C	-11.437224918926	102.386246217781	34.800635402559
S	-11.286501625899	104.088657355088	34.614128584073
H	-10.554810849998	105.220321299194	36.665733670442
H	-10.449478904308	102.943049784482	37.957967825208
C	-11.796277287054	101.235633371225	34.003939282825
C	-11.703052408742	100.109161585451	34.787265481395
S	-12.335047792739	100.896834855010	32.412129928025
C	-12.061796464519	98.937407733559	34.091947436787
C	-12.418582342028	99.180593956120	32.782045996650
H	-12.079758353743	97.945790465397	34.527338592852
C	-11.257575702474	100.458816689501	36.200221628939

C	-12.326673446520	100.080460198273	37.236542935586
H	-12.468256018585	98.996097959741	37.264976403053
H	-12.025372722475	100.406289533938	38.236357585133
H	-13.285462007050	100.545835371862	36.998019080839
C	-9.915776188772	99.796854884706	36.549058604196
H	-9.572635071844	100.119361983046	37.536397054331
H	-10.017460226805	98.707833631860	36.568016352496
H	-9.148428185987	100.057504039515	35.816930932539
C	-12.775447853250	98.182101052914	31.783510060354
C	-12.457354682758	96.850377267292	31.935627173314
C	-13.479266418855	98.479727112331	30.571902486076
H	-11.883372142242	96.534975664701	32.800826658705
C	-13.816859678850	97.424591853873	29.653335275660
C	-13.441917736682	96.069168538167	29.972705003667
C	-13.744328227805	94.935888045146	29.124444388558
C	-14.415909704524	94.881475622769	27.920067941531
S	-13.197546851661	93.352852220786	29.648151657088
C	-14.491616146770	93.569864463825	27.415916308863
H	-14.821072385541	95.766325613346	27.448389294234
C	-13.878947399723	92.648656129244	28.241103360192
C	-14.002685209268	91.344536005154	27.644533571933
C	-14.687248176439	91.453793311546	26.456713716008
S	-13.540760912170	89.728201048054	27.991213067069
C	-14.850534764594	90.215701246547	25.800238878278
C	-14.285869964449	89.168842997682	26.496375631483
H	-15.354860823521	90.066608843613	24.856090129911
C	-15.077034235107	92.898766307551	26.181904515283
C	-14.245773892866	87.758465425814	26.141687876959
C	-14.834996856118	87.234990096361	24.940491484843
C	-14.748968230691	85.824511205096	24.657057596736
C	-14.069434859247	84.954713659881	25.585502731170
N	-13.879683110270	99.677688057386	30.157889931782
N	-14.462139245286	97.852283930074	28.573693501085
N	-15.487348274430	87.909733288385	23.997574798868
N	-15.334422864034	85.476828272824	23.517013344937
S	-14.618113424034	99.464335209504	28.724221018073
S	-15.942303308026	86.834990447031	22.863599688682
C	-14.425638395350	93.421861136840	24.892537542419
H	-14.653398541264	94.482236583823	24.751193551042
H	-13.340232455915	93.305362611045	24.926143602320
H	-14.803544880782	92.876525040230	24.023010372349
C	-16.602871753221	93.067172538929	26.117575714183
H	-16.867222280779	94.121089050502	25.992622866363
H	-17.014728722885	92.512823453821	25.269426676425
H	-17.076757290226	92.700203145810	27.030588515052
C	-13.636010773958	86.805922614391	26.928679416967
H	-13.161910778673	87.104242704835	27.859542940479
C	-13.923844399408	83.528314989732	25.386617671175
C	-14.360591658340	82.725523196269	24.351942806284
S	-13.061750690374	82.616926503122	26.612643713620
C	-14.002769836567	81.378368398321	24.542370015978
H	-14.909819751880	83.122226499424	23.509157364736
C	-13.302081797781	81.176162185222	25.714607080586
C	-14.188108901529	80.075439224619	23.778926352835
C	-12.974697542357	79.778905490720	25.824125951054
C	-13.472961179333	79.117179417064	24.719693677227
C	-13.508259527049	80.126383919803	22.402013732386
C	-15.674369269419	79.715734888389	23.628301059465
S	-12.153151095536	78.742822931042	26.915949164731
C	-13.191120483912	77.738986370159	24.742430531869
H	-13.585169336141	79.157481806106	21.900446618755
H	-13.987970229157	80.874850336854	21.764853580222
H	-12.450496920917	80.381831083317	22.495370940319
H	-16.187705098500	80.458527248963	23.011046595663
H	-15.785783424058	78.740749700639	23.145412796263
H	-16.169441294563	79.675993568728	24.600988475548
C	-12.480412706973	77.374713635420	25.868317901762
H	-13.478851367073	77.020257012365	23.987341890709
C	-11.985804276027	76.076986637302	26.276427002296
C	-12.187162105845	74.871766268242	25.510838972100

C	-10.815145143255	74.826125367225	27.856485162077
C	-11.630621349700	73.635200978252	25.991918771896
N	-12.848485094684	74.741461397855	24.365947327641
C	-10.892376472237	73.606790845297	27.219120289761
H	-10.315816230195	74.895754451102	28.817302181381
N	-11.889182799188	72.603591382791	25.194667396040
S	-12.765139818982	73.172331238391	23.947561149857
C	-10.267027505950	72.409162567344	27.762975010882
C	-9.301022821181	72.365874143130	28.748287979746
S	-10.714360614768	70.792765155308	27.241103044909
C	-8.937036703316	71.052090080351	29.097724754070
H	-8.866481561708	73.258340563575	29.181238362014
C	-9.623012255450	70.104199549116	28.371105373483
C	-7.956000663401	70.402967907844	30.062784748175
C	-9.173751466838	68.795005927023	28.769567632857
C	-8.211865792595	68.935936281280	29.749507990660
C	-8.296047823925	70.735702138973	31.523685469609
C	-6.506728768365	70.802997427772	29.746200585724
S	-9.499715053057	67.157951429897	28.378089568318
C	-7.717725629774	67.700147608568	30.204749565353
H	-8.177846402189	71.806708303467	31.712159993987
H	-9.325028990875	70.456483765289	31.760357614855
H	-7.628952364214	70.198646510886	32.203930055469
H	-6.360262067718	71.875523152571	29.903864076129
H	-5.810634296822	70.268709779981	30.399045742224
H	-6.252889348676	70.570285438129	28.709763105275
C	-8.313002503414	66.634273098041	29.560370555574
H	-6.961312811453	67.561869472057	30.965059648375
C	-8.099594378051	65.211855413528	29.717371163761
C	-7.155296636732	64.645682548539	30.649646267325
C	-7.012166720167	63.213736310295	30.732394374761
N	-6.360669440364	65.306217878719	31.483442730405
C	-8.663098490904	63.052953726241	29.052675316754
C	-7.809145127781	62.367056440897	29.888496131669
N	-6.107350158433	62.838938896319	31.632773497788
S	-5.504281806884	64.193991756478	32.302735113986
H	-9.306555149856	62.499391048946	28.374519279811
C	-7.722369877678	60.915111143027	29.913189338674
C	-6.904515112058	60.114125255628	30.679969204862
S	-8.754435818777	59.946323300578	28.863555885452
C	-7.101838070984	58.739484520211	30.430127070532
H	-6.200902068650	60.529449388386	31.387440738273
C	-8.064518146686	58.499955388224	29.478307473249
C	-6.512012002837	57.436071403533	30.949397393653
C	-8.190554698100	57.078088865745	29.282405987078
C	-7.306456462444	56.436758178573	30.120832921732
C	-6.777065454000	57.258623578337	32.452333398576
C	-5.006641271212	57.343783665166	30.656617732477
S	-9.139000435408	55.990216122896	28.356102067653
C	-7.381183607642	55.035104358888	30.011278568392
H	-6.412651791253	56.285798675585	32.795001210604
H	-6.260594685592	58.033214744959	33.026303544637
H	-7.844894513217	57.322488358079	32.672408569731
H	-4.462087664561	58.120185384153	31.201440088557
H	-4.612698415120	56.372743746275	30.970381958290
H	-4.806197049592	57.467703143070	29.590201543685
C	-8.316529353670	54.619476935770	29.085613012030
H	-6.789817799193	54.341115236224	30.595774037448
C	-8.593540630848	53.244459266422	28.693872790428
C	-7.713264578357	52.217609361957	28.956778076212
C	-9.784642130181	52.832844417515	28.012933486522
H	-6.761327690787	52.439012542837	29.428329674975
C	-9.990824712610	51.445609607433	27.690951518562
N	-10.781473966185	53.614225153831	27.609917024815
C	-8.986959652143	50.480439338070	28.066718433313
N	-11.135168585695	51.216795593821	27.056293042415
S	-11.877177555033	52.653970228971	26.885811753123
C	-9.106452472628	49.062524937917	27.804966344020
C	-10.126600991770	48.353607174502	27.203221122791
S	-7.785594655750	48.021473911189	28.306396536740

C	-9.849438775103	46.975770903995	27.143733422005
H	-11.019985460340	48.836942365180	26.831992081494
C	-8.626453144780	46.656333959088	27.698794372041
C	-10.556695231729	45.734382769121	26.620419433182
C	-8.431215275273	45.234205023636	27.591420656495
C	-9.525302639982	44.673551307153	26.974939928083
C	-10.785559327987	45.811309369949	25.103071380141
C	-11.886696793298	45.492321876594	27.350651842993
S	-7.242051768049	44.067801696709	28.007394078145
C	-9.417079725542	43.274700652120	26.826984204198
H	-11.473509404772	46.626149637743	24.860271720041
H	-9.846661769097	45.984892064473	24.572855248142
H	-11.222341038894	44.879159967898	24.733559539010
H	-12.590741077078	46.303172426113	27.143002381570
H	-12.342556566496	44.555697599561	27.017189377190
H	-11.736688017075	45.435847001295	28.430992865297
C	-8.235111232643	42.778836269465	27.332834430891
H	-10.157638211663	42.631553912111	26.373276737958
C	-7.765007184037	41.401928478188	27.356485243222
C	-8.547823793900	40.299286889369	26.871494961824
C	-6.528385699999	41.040443759707	27.844451997290
C	-8.012279336884	38.962414542066	26.927654571552
N	-9.769863529386	40.341815257875	26.347433745261
H	-5.861059647155	41.806090702638	28.230379802034
C	-6.692896155341	38.748420295022	27.471368649457
N	-8.838737214280	38.040539336883	26.448160016903
S	-10.184307256280	38.814609450061	25.966976869773
C	-6.069908252413	37.446478873819	27.565980119309
C	-6.544306944135	36.204276464228	27.194328670948
S	-4.455314374200	37.358175615516	28.248623018306
C	-5.614298285820	35.182834212282	27.457567593062
H	-7.523334070965	36.071394620617	26.754863959246
C	-4.448289140759	35.658711761514	28.023116254001
C	-5.561051784914	33.673181001911	27.274155188524
C	-3.556628029976	34.549701770440	28.245896424804
C	-4.171005954842	33.391123875838	27.824812260202
C	-5.672892996447	33.280042771672	25.793145870001
C	-6.647487074349	32.966436029794	28.099347169417
S	-1.999320727040	34.273792232858	28.910124032436
C	-3.369804742393	32.251875915400	28.028136127283
H	-5.557301392912	32.199072943636	25.672097815222
H	-6.652351985768	33.560252259761	25.395193898199
H	-4.905010397457	33.776787270973	25.196154379756
H	-7.642612350790	33.242445398245	27.739108855562
H	-6.548759075984	31.880175146501	28.016188332359
H	-6.577018363953	33.237985412626	29.154846526419
C	-2.148271526304	32.552413175849	28.596247685535
H	-3.669736562162	31.239190032642	27.788785200617
C	-1.072320986666	31.611646678260	28.876473391221
C	-1.018612315888	30.367218611562	28.287521665454
C	0.011272979954	31.881505746181	29.773360754363
H	-1.771336090495	30.089782211473	27.556845316555
C	1.016732101969	30.880934447362	30.014209392483
N	0.224653347079	33.006568286997	30.448150188573
C	0.913879233020	29.607390043782	29.345569410934
N	1.959621168145	31.275557354329	30.862933979028
S	1.589031542512	32.795130868064	31.308143390588
C	1.860906761490	28.528534910933	29.531427269537
C	2.981078742897	28.460025467316	30.335148560247
S	1.603620014684	27.048078685555	28.626745669046
C	3.633863694951	27.218640860664	30.225100957805
H	3.289886121481	29.285392138202	30.961979335252
C	3.003683137210	26.364146275301	29.342679599611
C	4.866754469758	26.569557884466	30.836001957049
C	3.735519309812	25.125731747932	29.293527428703
C	4.817944635438	25.214563489291	30.145929669393
C	4.733869251678	26.431512700364	32.360565140276
C	6.147239224220	27.339653419282	30.478010869386
S	3.634411994426	23.615251359021	28.488531514438
C	5.593296069467	24.040917189433	30.159153995633

H	4.693315915653	27.417185928590	32.832827995323
H	3.826722889294	25.885061946453	32.627595959624
H	5.592710119643	25.894867887042	32.773721829183
H	6.130349977837	28.339302592926	30.921479700033
H	7.028667406510	26.816361785471	30.859467862385
H	6.252160083853	27.445555675919	29.396073328001
C	5.085483266926	23.075341285817	29.313107420816
H	6.485814397383	23.877757691214	30.747397695659
C	5.566257955054	21.740333207325	29.027228105846
C	6.744036698674	21.170062024047	29.634012034735
C	7.143196357290	19.830082066951	29.284789219872
N	7.548693875321	21.748822091843	30.517663127064
C	5.273038375667	19.756469678865	27.845270644277
C	6.366180824716	19.078303981882	28.338716837880
N	8.244044377697	19.439315425684	29.921541880878
S	8.714541217397	20.673716803169	30.871875600699
H	4.624841845469	19.271840842677	27.120513807479
C	6.701946945314	17.720936998572	27.936472254693
C	7.776159417713	16.949652065320	28.324319509080
S	5.669222728871	16.850193204426	26.806124417314
C	7.773521641892	15.674480794591	27.720758330793
H	8.519767916223	17.314982823060	29.018251130836
C	6.701786506738	15.481011572584	26.880762302788
C	8.664625485589	14.441972132953	27.763021074658
C	6.778227682334	14.160466105441	26.313260732360
C	7.905799599276	13.534502724095	26.805824918338
C	8.729545963647	13.843362993632	29.176588030748
C	10.078243650115	14.751236213308	27.246708244629
S	5.882981287615	13.179923262753	25.228600371469
C	8.063343310473	12.230563510597	26.302348487589
H	9.313362831129	12.918500557460	29.174620481148
H	9.207624318203	14.543020287861	29.868082070152
H	7.729637307412	13.617391816384	29.553336712646
H	10.580660109833	15.464315098970	27.906515461928
H	10.681651087341	13.839637807102	27.213075966558
H	10.044373694133	15.177871085155	26.241890026415
C	7.048907746167	11.884243572944	25.432497578911
H	8.868504594475	11.550571614893	26.545030279708
C	6.829178912427	10.655697091695	24.700848174380
C	7.706398187570	9.513679552689	24.782454611547
C	5.492894316150	9.466638237522	23.206591554418
C	7.408986257357	8.344081866287	23.998871961628
N	8.805101975037	9.388971338859	25.518582571921
C	6.251793635620	8.317814887911	23.155133416358
H	4.571675718185	9.523637115508	22.635970329636
N	8.293673617797	7.366532867954	24.167173855923
S	9.400396612148	7.902922320626	25.232339490048
C	5.890111011379	7.188971024942	22.308487896077
C	4.993910014096	7.205253756382	21.259153655661
S	6.541872827442	5.576937621469	22.560986373880
C	4.812722313970	5.936488375465	20.676014842296
H	4.505110681663	8.110859837933	20.921879165922
C	5.578249101809	4.965455979461	21.281079338619
C	4.000111910173	5.359180709654	19.525814806475
C	5.351477442605	3.712261436939	20.606821385515
C	4.443358355132	3.904683222342	19.592400481168
C	4.384342421664	6.004234655362	18.185392989577
C	2.490474166381	5.499238142530	19.774691487616
S	5.875605621378	2.082722257849	20.720778700006
C	4.154314203824	2.720134738361	18.882422014390
H	4.126145896602	7.067187464410	18.182480450413
H	5.456188516439	5.910637169728	17.997862984647
H	3.847956560963	5.525294063582	17.361325898982
H	2.200556684450	6.553770636499	19.794698857105
H	1.923283921823	5.009094617107	18.978320293310
H	2.206349265521	5.046751115056	20.727227676683
C	4.849578292651	1.632175842384	19.362062179065
H	3.469664791495	2.637104926695	18.049996987049
C	4.820189197723	0.255651052080	18.886824649363
C	3.957446822484	-0.179864127014	17.820746741157

C	5.613890827298	-0.734422899238	19.420328183914
C	3.991951108190	-1.558315449347	17.418703491456
N	3.087421951345	0.541042330865	17.116429445853
H	6.300362733117	-0.493245592228	20.226563908683
C	4.875102393644	-2.460248454814	18.081224285012
N	3.155557765115	-1.852932482178	16.426744027414
S	2.391824500461	-0.471587076510	16.046794843160
H	4.909631842429	-3.505916593900	17.787231103371
N	-12.783411998452	95.844528825508	31.087577146655
N	-13.548878045534	85.479298376853	26.671453263073
N	-11.319296867377	76.000523086783	27.406818894590
N	-8.805894360301	64.397016082858	28.966940882134
N	-7.901384967883	50.905752283779	28.674071021510
N	-6.015453739212	39.788420438439	27.902719994711
N	-0.087516426409	29.410062077554	28.517323546865
N	4.888742594869	21.014449829741	28.167129519741
N	5.769669571114	10.581010452253	23.926077827889
N	5.647633805773	-2.047147697248	19.041111532635

Oligomer 3 in solvent

Atom	X	Y	Z
C	-56.711741840215	-3.828897809193	1.076408161232
C	-55.851833254789	-4.876139642241	1.242436791790
C	-54.495420146889	-4.462920651064	1.161810160279
C	-54.365735319271	-3.113620848797	0.936773989215
S	-55.887974620609	-2.319670633636	0.818565468857
H	-57.792096640140	-3.845401096715	1.088896752843
H	-56.185446193576	-5.892420347080	1.414059555042
C	-52.960977643231	-2.786119345973	0.869565374470
C	-52.225358501100	-3.931496502009	1.052447448573
S	-51.974131800564	-1.401221006247	0.629891843483
C	-50.831896797705	-3.704923849996	1.002466060795
C	-50.516030542199	-2.382584537641	0.781929163983
H	-50.070377415215	-4.462929662405	1.119731635386
C	-53.133547234911	-5.135193723139	1.260197523264
C	-52.941902881740	-6.183674419558	0.153182622409
H	-51.932860318351	-6.603167538961	0.194425001414
H	-53.654546102593	-7.003907628883	0.276709081258
H	-53.091619575482	-5.743346049009	-0.835320952344
C	-52.916698351404	-5.772335925347	2.641625601025
H	-53.625476749370	-6.589702300311	2.801184769074
H	-51.905603518248	-6.181743015605	2.718842280817
H	-53.052586717881	-5.037993594434	3.438948722554
C	-49.201404664823	-1.767946173782	0.673225281523
C	-49.017706606844	-0.410223445376	0.520144466791
C	-47.979907743778	-2.520531499049	0.715498119851
H	-49.885360029898	0.242915645558	0.483289891115
C	-46.721944087736	-1.835147235728	0.598041579194
C	-46.692396306499	-0.405957301411	0.437950740930
C	-45.466797383509	0.365566060819	0.309242680008
C	-45.415838766915	1.737580162867	0.159385399467
S	-43.857544524315	-0.340956014380	0.323684252103
C	-44.101331221000	2.222158870935	0.056682586418
H	-46.319226565678	2.332424382570	0.129820451453
C	-43.161433362201	1.214282619078	0.129053519612
C	-41.850188154185	1.796466167831	0.015415727146
C	-41.974567698139	3.158895632714	-0.126905940791
S	-40.212467866458	1.279473989225	0.007196103128
C	-40.730358057421	3.814452521667	-0.247515573936
C	-39.664239438520	2.942601128833	-0.196561620256
H	-40.593611816898	4.879680405906	-0.368127462834
C	-43.436817771959	3.580290028808	-0.115535970642
C	-38.242302123382	3.238481767176	-0.291472202045
C	-37.725897772543	4.572116643676	-0.424919773874
C	-36.305366593383	4.782478952280	-0.515565880987
C	-35.416184381242	3.649856935656	-0.470927400053
N	-47.834916466935	-3.836831852449	0.855336356157
N	-45.669644737441	-2.643558718082	0.652206747489
N	-38.417475783575	5.708166610844	-0.479754126441
N	-35.966850593053	6.061997155339	-0.635739718852

S	-46.239331058797	-4.152632772086	0.837517542400
S	-37.344427115503	6.920261134706	-0.632915308263
C	-43.745517237175	4.513924846461	1.065427510782
H	-44.813945951134	4.743694316156	1.100115189184
H	-43.460644306671	4.055149466015	2.015020775397
H	-43.199909638618	5.455643894147	0.959895829251
C	-43.842444070732	4.244007643012	-1.440877313406
H	-44.911599325439	4.473165432520	-1.440709340707
H	-43.295371268677	5.180299602194	-1.581296925349
H	-43.630238489313	3.590070828745	-2.289811762942
C	-37.273025832341	2.259816410902	-0.263163466829
H	-37.565839309193	1.218206495833	-0.166914449142
C	-33.974304619474	3.759356539501	-0.557393994481
C	-33.171735268296	4.873922527992	-0.689159142865
S	-33.037008702345	2.275727176901	-0.493214562979
C	-31.804830463334	4.539326705974	-0.739230351714
H	-33.578035002892	5.874336973759	-0.745257033424
C	-31.586494969050	3.180127944131	-0.645313727677
C	-30.494746551854	5.300814248104	-0.873521603196
C	-30.170398801560	2.932776173092	-0.707165053900
C	-29.513291939547	4.139057128040	-0.839233335989
C	-30.276014357000	6.261363423457	0.306086984174
C	-30.420274250143	6.064922517019	-2.204975605425
S	-29.105009699998	1.588174462470	-0.665663389738
C	-28.114859802782	3.989705943606	-0.909686024053
H	-29.296610311637	6.741511003393	0.230052843429
H	-31.038254297854	7.045270977665	0.305193334054
H	-30.327292006919	5.731871034886	1.260191992039
H	-31.185933827637	6.844738819306	-2.239866710682
H	-29.443816526747	6.544364494882	-2.315552597984
H	-30.573030738615	5.393846670333	-3.053284937013
C	-27.729616089883	2.667224004665	-0.829785755733
H	-27.399378013566	4.793730366165	-1.014055690618
C	-26.407843008670	2.075906818298	-0.865064145389
C	-25.195791740069	2.844344115488	-0.988298191813
C	-25.119799539317	0.133145143283	-0.805463334226
C	-23.933334813730	2.159611415329	-1.034492105010
N	-25.081042535339	4.166320471051	-1.070134466795
C	-23.886971441853	0.730703319567	-0.953460365503
H	-25.181310084875	-0.944106570059	-0.692818042117
N	-22.898349414709	2.987314176205	-1.149826328972
S	-23.496473202877	4.499176351918	-1.192767513107
C	-22.658295385536	-0.049427446224	-1.023151392396
C	-22.560932927817	-1.404144193044	-1.267213995073
S	-21.074527045973	0.661789052532	-0.744760771867
C	-21.234186184162	-1.873835401152	-1.212585594851
H	-23.420213959380	-2.023144405599	-1.493027376142
C	-20.329795226772	-0.873415230023	-0.934181576619
C	-20.535642417468	-3.211546161011	-1.405114401169
C	-19.003472097858	-1.434398621980	-0.923360196245
C	-19.090998530148	-2.785579616766	-1.189352444766
C	-20.755805399192	-3.764246041330	-2.822010701610
C	-20.984883689815	-4.239658884094	-0.354907931488
S	-17.390419719473	-0.903858272546	-0.673844272646
C	-17.834703628029	-3.420597728804	-1.195323311555
H	-21.812779209970	-3.993917079616	-2.982795070369
H	-20.440804395544	-3.042728721842	-3.579337511885
H	-20.184168348459	-4.685411208342	-2.964645428637
H	-22.045479666658	-4.474754925683	-0.479798907054
H	-20.417604286691	-5.168103360429	-0.463384764243
H	-20.833172500077	-3.859592913360	0.657953155956
C	-16.804951873654	-2.540062187633	-0.933698702731
H	-17.661061963432	-4.471474380330	-1.381519440310
C	-15.377613979039	-2.767801834924	-0.853651967890
C	-14.766317154099	-4.058737596800	-1.047391344969
C	-13.335975105603	-4.185901295252	-0.958174937766
N	-15.383099313908	-5.204871200283	-1.315458103341
C	-13.257012829606	-1.869407313524	-0.504178285977
C	-12.533504817766	-3.028966407260	-0.675155660517
N	-12.916765899077	-5.432177405579	-1.164814565791

S	-14.233958273617	-6.344593982206	-1.443099852517
H	-12.737268275385	-0.944284703320	-0.271344265531
C	-11.081859955472	-3.076097531464	-0.580787246140
C	-10.256498802933	-4.178889490990	-0.555728009840
S	-10.149990929914	-1.583024145950	-0.477108115525
C	-8.891925244960	-3.834235671531	-0.446990024827
H	-10.644178718248	-5.185906288038	-0.616613472830
C	-8.685109627395	-2.475704594213	-0.393453778557
C	-7.573760556391	-4.590944874822	-0.376928842328
C	-7.271232204132	-2.222434117803	-0.288556800325
C	-6.602605206284	-3.423919189279	-0.276575367803
C	-7.330776075804	-5.418054297547	-1.649060421431
C	-7.505190811887	-5.490943068487	0.866784963735
S	-6.209535982142	-0.876735091224	-0.177932095230
C	-5.202575580627	-3.273146501277	-0.177975793098
H	-6.346661690590	-5.893331594765	-1.612784165611
H	-8.083931877584	-6.205347173570	-1.741905653275
H	-7.377240310104	-4.789770039322	-2.541524345963
H	-8.261656550374	-6.278413760811	0.809431706351
H	-6.524086876237	-5.968298155655	0.938865917445
H	-7.675905883607	-4.914866796141	1.779126097816
C	-4.813606373434	-1.952749462783	-0.117463234316
H	-4.486739006646	-4.082450405229	-0.150965277928
C	-3.468192216162	-1.406579810995	-0.021890132271
C	-3.202898622333	-0.054358408937	-0.064082761603
C	-2.299219488304	-2.226640918372	0.122506657822
H	-4.026173370129	0.645582564060	-0.178468525058
C	-1.005819288633	-1.605583490402	0.207928669398
N	-2.235607876744	-3.554705054548	0.194169853521
C	-0.889294478531	-0.172819017076	0.151105529158
N	-0.008277224229	-2.472353771823	0.339550443918
S	-0.667345477209	-3.956193390258	0.353973254396
C	0.376762446439	0.536376338297	0.229627921998
C	0.510716568395	1.910321160774	0.178016125321
S	1.936487427389	-0.256511301574	0.397310458348
C	1.848457710644	2.327359163150	0.271191982405
H	-0.352252365922	2.554955526364	0.075072944194
C	2.723689785965	1.267119343914	0.392521463145
C	2.592091938152	3.655012947071	0.270769519464
C	4.064891271556	1.782660882781	0.477423245046
C	4.022820976745	3.155937082213	0.410434724844
C	2.182137597225	4.532353402229	1.463975559870
C	2.386529251259	4.416336828030	-1.048116393534
S	5.665383473957	1.179533446770	0.635649435415
C	5.301762769887	3.749209457165	0.484351051680
H	1.130589651900	4.820466786442	1.381515345926
H	2.320972184152	4.001692749590	2.408682152387
H	2.782778218559	5.445691145462	1.489815339267
H	1.336946336376	4.699702973400	-1.164677125359
H	2.987198666122	5.329974831906	-1.059173580079
H	2.675174996556	3.803678559804	-1.905260225137
C	6.310982992618	2.818754383074	0.602300704651
H	5.501585647385	4.810938421444	0.454032242398
C	7.747248247584	3.041276940833	0.684631699370
C	8.327519178177	4.340421606936	0.877608586629
C	8.667464321615	2.021626606627	0.582170981583
C	9.757582971622	4.479503892824	0.951343974552
N	7.690263377048	5.501530430996	1.009278759280
H	8.322630074408	1.004325173403	0.420028672704
C	10.591692307507	3.310176096417	0.831014008737
N	10.157830907106	5.733362417345	1.134114040037
S	8.822000759948	6.653017033999	1.205994056743
C	12.037520815248	3.349964321968	0.888867735391
C	12.894936811208	4.417838879230	1.059736971712
S	12.904956321898	1.832029971614	0.714342675097
C	14.245622886518	4.021278548495	1.049974397200
H	12.536857918568	5.430690737961	1.182867928960
C	14.398843482403	2.661165669034	0.872815217126
C	15.594169545484	4.713560702026	1.182230301413
C	15.804475129278	2.352234136032	0.873002605095

C	16.520037877549	3.513653158409	1.048601410933
C	15.749752383945	5.395043842314	2.550781067567
C	15.821210865429	5.728832761846	0.051079910335
S	16.815775298488	0.972785801187	0.713229563576
C	17.915678758379	3.303197099384	1.058861047472
H	16.753468296578	5.816247857277	2.654586355158
H	15.028323096440	6.210067465984	2.655200437532
H	15.587967228818	4.684929135673	3.364961129144
H	15.097137323582	6.545147213851	0.121128950753
H	16.824093451923	6.159008592384	0.120148573972
H	15.715112251068	5.256979407041	-0.928410128339
C	18.252704020776	1.977472193985	0.891855456982
H	18.664141391219	4.073273321766	1.180474882587
C	19.578071661091	1.377279354394	0.853756454213
C	19.782018245772	0.015311214161	0.790011634552
C	20.787530464431	2.149305032802	0.878185841566
H	18.924057597923	-0.651363214445	0.777269048921
C	22.056386846656	1.476359006812	0.830942591164
N	20.911764502843	3.473566523523	0.940958893112
C	22.107794295848	0.040840504568	0.758266041696
N	23.096543329194	2.301712962789	0.859722352837
S	22.503016363717	3.810471001803	0.940211186206
C	23.345755563209	-0.719853296733	0.702772760631
C	23.415634976420	-2.097982784915	0.640657481340
S	24.944155548872	0.008659568280	0.703784481763
C	24.737762486720	-2.569606491880	0.593836379940
H	22.520567509713	-2.705774367047	0.631240502662
C	25.663495610700	-1.546166112811	0.620307565873
C	25.420256248992	-3.927403866144	0.521184099435
C	26.985193234449	-2.112661974677	0.569029833442
C	26.876753350572	-3.487272666992	0.511068881559
C	25.048099520126	-4.678825190988	-0.766682129103
C	25.097408080467	-4.787385302195	1.753298350061
S	28.611887732401	-1.565866187873	0.557896394231
C	28.129488886557	-4.127905043847	0.455853605337
H	23.981709143336	-4.920333407779	-0.772368942337
H	25.270339520460	-4.077961484806	-1.651481911463
H	25.607801061735	-5.615543893250	-0.836054716089
H	24.030666879488	-5.025558991740	1.782599888400
H	25.653319623963	-5.728291125727	1.717862161520
H	25.359808645200	-4.266576549443	2.676997040181
C	29.174622227108	-3.227232464281	0.472939667785
H	28.289474287825	-5.196041495861	0.404889421184
C	30.604682069468	-3.452621910220	0.429404643284
C	31.203191041565	-4.761414913980	0.359941899519
C	32.635944403013	-4.883312912959	0.312674571961
N	30.571750203875	-5.930422154223	0.328876056798
C	32.741085906316	-2.525747539226	0.413557718739
C	33.453034908201	-3.702570803949	0.337497123986
N	33.041875851531	-6.149241378331	0.245793753885
S	31.711101505473	-7.083612149028	0.245699151727
H	33.273002219647	-1.579311858623	0.450275729257
C	34.907216526681	-3.742224847389	0.285237105512
C	35.740780454656	-4.837680078495	0.348763158401
S	35.829250443586	-2.248991281139	0.124804474455
C	37.105734955250	-4.485474154210	0.276569221005
H	35.358401857444	-5.844126027869	0.442406458749
C	37.303433085596	-3.129647780614	0.155768713997
C	38.430592333943	-5.233141254707	0.296210421113
C	38.716221501065	-2.864469566159	0.085509825076
C	39.394807103045	-4.063472839692	0.164551816361
C	38.548135100459	-6.200501107472	-0.892156633645
C	38.633578407393	-5.986364937744	1.620266563924
S	39.759638898687	-1.510619081569	-0.065029827121
C	40.791968142906	-3.900506583147	0.104144658279
H	39.533300205429	-6.674731283452	-0.901946686768
H	37.793732767334	-6.988468982116	-0.817822279136
H	38.407925545959	-5.677511397194	-1.840918937100
H	37.883417182054	-6.774346329232	1.729757679850
H	39.621707032405	-6.453915106854	1.645185013535

H	38.550660071296	-5.310326498366	2.474340384599
C	41.156178160023	-2.575488322340	-0.021333180982
H	41.521825471961	-4.697285221963	0.147404882717
C	42.468462674536	-1.971600541017	-0.115866401569
C	43.694904264539	-2.727974314434	-0.098159200572
C	43.726594523228	-0.018895400931	-0.320973876973
C	44.949425952295	-2.031863458950	-0.180344477689
N	43.829804126247	-4.047410043126	-0.009305750047
C	44.973194910309	-0.603953882429	-0.284912352667
H	43.645577206403	1.056292498843	-0.442129249289
N	45.999057432201	-2.848662569058	-0.151332318052
S	45.422422304467	-4.365023786389	-0.031398516845
C	46.194887865703	0.187999524465	-0.346159147277
C	46.309898850419	1.538820731844	-0.092497731792
S	47.737703242310	-0.500268711313	-0.834835055918
C	47.615388906955	2.025017926060	-0.306575938315
H	45.479490731665	2.143111127811	0.251073123437
C	48.485389390299	1.040143807491	-0.715612702601
C	48.322782136472	3.366285458023	-0.178247514493
C	49.794014788840	1.620357199269	-0.884041735569
C	49.732228980538	2.961257106078	-0.584838067595
C	48.281468275132	3.889714911002	1.266074606367
C	47.734973061927	4.410003434391	-1.140977995967
S	51.369899509517	1.120377088803	-1.346229964992
C	50.975976876742	3.613507998175	-0.729029752571
H	47.251973644232	4.106000447591	1.564692579672
H	48.695651892345	3.156901499372	1.962332392150
H	48.859799107949	4.813597053943	1.353375526375
H	46.697292508908	4.631544543809	-0.876586822098
H	48.303921972618	5.342233462489	-1.086767271076
H	47.758613229210	4.051306654302	-2.172548667476
C	51.975885383626	2.761649956378	-1.142093601652
H	51.157297628888	4.662560610734	-0.541243648301
C	53.374854653111	3.068088069404	-1.412105642460
C	53.984413233527	4.310408753455	-1.022138258597
C	54.217464819239	2.191733121456	-2.057063166261
C	55.370776110690	4.525702757181	-1.319027982647
N	53.425633386520	5.341064097691	-0.389456652217
H	53.832992721788	1.236505143896	-2.401981688810
C	56.111503166396	3.509034846746	-1.987737185960
N	55.832019251333	5.706471123966	-0.910462402222
S	54.584944650771	6.468290562176	-0.205471624194
H	57.163718490279	3.658608367459	-2.214294727505
N	-47.835686611158	0.243803741313	0.405996830786
N	-35.933566759238	2.447431297000	-0.346954815416
N	-26.318373107443	0.766355200717	-0.775852541813
N	-14.603408219915	-1.737376465447	-0.591714937347
N	-1.987563195491	0.539729150822	0.019220110989
N	10.015680518924	2.140854969596	0.654748830020
N	20.974457139717	-0.626921943595	0.741511798587
N	31.392293418374	-2.399914141307	0.453782579038
N	42.536324757521	-0.662308423929	-0.227652259146
N	55.541839270534	2.391725366956	-2.3349783559
